# Real-world safety of Symdeko: insights from the food and drug administration adverse event reporting system

**DOI:** 10.3389/fmed.2025.1681985

**Published:** 2025-10-30

**Authors:** Su Wei, Lixi Zhang, Cuiping Liu, Xu Qi

**Affiliations:** Department of Respiratory and Critical Care Medicine, The First Affiliated Hospital of Nanjing Medical University, Nanjing, China

**Keywords:** CFTR modulator, pharmacovigilance, FAERS, safety signal, adverse event

## Abstract

**Background:**

Symdeko, a cystic fibrosis transmembrane conductance regulator (CFTR), is widely utilized for treating cystic fibrosis (CF). Although its safety has been validated in numerous clinical trials, the real-world safety profile still needs further investagation.

**Methods:**

This study analyzed adverse event (AE) reports related to Symdeko from the FDA Adverse Event Reporting System (FAERS) spanning Q1 2018 to Q3 2024. Four disproportionality analysis methods were employed: Reporting Odds Ratio (ROR), Proportional Reporting Ratio (PRR), Multi-Item Gamma Poisson Shrinker (MGPS), and Bayesian Confidence Propagation Neural Network (BCPNN). A descriptive analysis of the time to onset (TTO) of AEs was conducted, and the Weibull distribution was used to predict temporal variations in AEs. Sensitivity analysis was also implemented to refine the results.

**Results:**

This study included 5,245 AE reports. Of these, 54.9% were from females, 41.3% from males, and 62.3% from healthcare professionals. The study confirmed several known AEs, including headache, nausea, and elevated liver enzymes, and identified novel signals, such as anxiety, depression, suicidal ideation, and renal stones. In females, particular attention should be paid to dizziness, anxiety, and lower respiratory infections. In males, the occurrence of nausea and abdominal pain should be noted. For patients aged 18 and older, kidney stones should be monitored. The median time to onset (TTO) of AEs was 188 days, with 51.3% of AEs occurring after six months of treatment initiation. Sensitivity analysis further confirmed that headache, nausea, anxiety, depression, suicidal ideation, and renal stones continued to exhibit positive signals.

**Conclusion:**

The study confirmed several known AEs associated with Symdeko and identified various novel signals, providing preliminary real-world safety evidence for Symdeko. These findings underscore the importance of ongoing monitoring and management for patients treated with Symdeko. Future larger-scale prospective studies are necessary to validate these findings and enhance patient management strategies.

## 1 Introduction

Cystic fibrosis (CF) is an autosomal recessive genetic disorder caused by mutations in the cystic fibrosis transmembrane conductance regulator (CFTR) gene, affecting over 89,000 individuals worldwide (1). CF can affect multiple organs, with the respiratory system being the most severely impacted (2). Common symptoms include cough, shortness of breath, sputum production, and difficulty breathing (3, 4). Traditional treatments primarily alleviate the symptoms of CF without altering disease progression. In recent years, there have been significant advancements in CF treatment, driven by the introduction of novel therapeutics, including CFTR modulators. CFTR is a protein that regulates chloride ion flow and is essential for maintaining the normal function of the respiratory system and other organs (5). Mutations in the CFTR gene lead to defective protein, disrupt ion flow, and consequently cause the thick, viscous mucus characteristic of CF (6). To address this problem, researchers have developed multiple CFTR modulators, including correctors, potentiators, and the investigational amplifiers and stabilizers. Amplifiers enhance function by increasing the synthesis of CFTR protein, whereas stabilizers help maintain CFTR protein stability and ensure better performance at the cell membrane (7). Although these approaches remain in clinical trials, they offer new possibilities for CF treatment.

On 12 February 2018, tezacaftor/ivacaftor (Symdeko) was first approved by the United States Food and Drug Administration (FDA) (8). Symdeko is a novel CFTR modulator combination containing two components that work synergistically through distinct mechanisms. Tezacaftor is a CFTR corrector that increases the amount of functional CFTR proteins on the cell surface (9). Ivacaftor is a CFTR potentiator that enhances the activity of CFTR by prolonging the opening time of the ion channel at the cell membrane. This combination therapy improves CFTR function, significantly benefiting CF patients (10, 11). Several randomized clinical trials (RCTs) have demonstrated that the predicted FEV1 percentage (ppFEV1) of subjects in the tezacaftor/ivacaftor group was 3.75%–6.80% higher than baseline levels. For example, in the EVOLVE trial, tezacaftor/ivacaftor showed a mean increase in ppFEV1 of 4.5%, while the EXCEL trial also confirmed significant improvements in lung function. In addition, the therapy also showed a positive effect in reducing the incidence of acute pulmonary exacerbations (9, 12). In terms of safety, compared with another dual therapy, lumacaftor/ivacaftor, tezacaftor/ivacaftor demonstrated better tolerability, particularly characterized by a lower incidence of respiratory AEs, such as cough and dyspnea (13). Overall, this combination therapy exhibits favorable efficacy and safety in CF patients. Although Symdeko is no longer the newest CFTR modulator therapy, it is still widely used. Previous RCTs had limitations, such as small sample sizes, short follow-up periods, and strict inclusion/exclusion criteria, which restricted the generalizability of Symdeko’s safety profile. Analyzing real-world AE reports related to Symdeko helps to reveal its safety under broader conditions, providing initial real-world safety insights for clinicians and regulatory agencies.

The FDA Adverse Event Reporting System (FAERS) collects AE reports and medication error information spontaneously reported by clinicians, pharmacists, nurses, and consumers, primarily for post-market drug safety surveillance (14). Given the large volume of real-world AE reports in the FAERS database and its accessibility, an increasing number of researchers are utilizing FAERS to assess drug safety (15, 16). This study aims to comprehensively assess the real-world safety of Symdeko through disproportionality analysis using data from the FAERS database, providing preliminary safety insights for healthcare professionals.

## 2 Materials and methods

### 2.1 Data sources and data extraction

Food and Drug Administration Adverse Event Reporting System collects AE reports that are spontaneously submitted by clinicians, pharmacists, nurses, and consumers. The relationship between drugs and AE reports is classified as primary suspect (PS), secondary suspect (SS), concomitant (C), and interaction (I). In this study, the trade name “Symdeko” was used as the search term, and AE reports from Q1 2018 to Q3 2024 in which “Symdeko” was listed as the PS drug were extracted. To ensure the data accuracy, reports with misspelled drug names were excluded. The study period was chosen from Q1 2018, the time when Symdeko was first approved, to Q3 2024, as this represents the latest available data from the FAERS database at the time of conducting this study. This time frame ensures that the most recent and relevant data was included in the analysis.

### 2.2 Report deduplication and AE standardization

**FIGURE 1:**
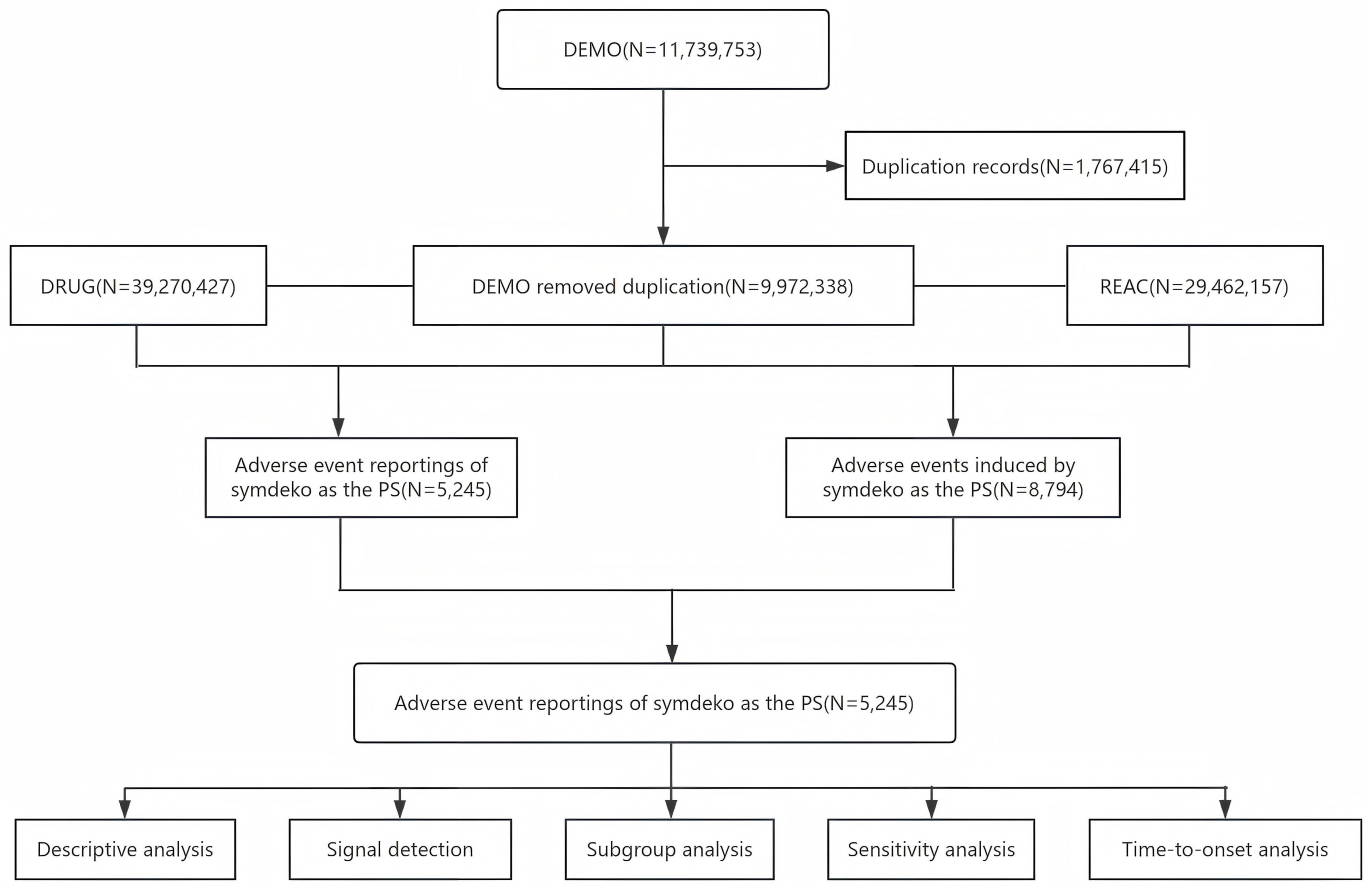
Flowchart depicting the process of analyzing adverse events (AEs) related to Symdeko using the Food and Drug Administration (FDA) Adverse Event Reporting System. DEMO, demographics; DRUG, drug information; REAC, adverse events; PS, primary suspected.

Report deduplication follows FDA guidelines (17), primarily accomplished by using three parameters: case identifier (CASEID), FDA receipt date (FDA_DT), and primary identifier (PRIMARYID). Specifically, when two reports share the same CASEID, the report with the later FDA_DT is retained. When both CASEID and FDA_DT match, the report with the higher PRIMARYID value is kept. Deduplication was performed using R software. AEs are standardized using the Medical Dictionary for Regulatory Activities (MedDRA, version 27.0) and subsequently mapped primarily to system organ class (SOC) and preferred term (PT) levels. A flowchart depicting the data source, extraction, and processing procedures of this study is shown in Figure 1.

### 2.3 Statistical analysis

#### 2.3.1 Disproportionality analysis

This study used four disproportionality approaches: Reporting Odds Ratio (ROR) (18), Proportional Reporting Ratio (PRR) (19), Bayesian Confidence Propagation Neural Network (BCPNN) (20), and Multi-Item Gamma Poisson Shrinkage (MGPS) (21). ROR evaluates the association between a drug and an AE by calculating the reporting odds ratio and is among the most widely used tools in pharmacovigilance, although sparse counts can inflate false positive findings. PRR compares the event reporting proportion for the index drug with that of a comparator group and can reflect differences in drug exposure, yet small denominators may yield unstable estimates. BCPNN integrates Bayesian inference with neural network modeling, accommodates high dimensional and complex probability structures, and quantifies uncertainty to produce more stable and reliable results. MGPS applies empirical Bayes shrinkage to reduce spurious signals and shows robustness for rare events and limited sample sizes, thereby improving detection accuracy. To more comprehensively detect potential adverse reactions, any AE that met the predefined threshold in any single method was deemed a positive signal. All algorithms are based on 2 × 2 contingency tables, as detailed in Supplementary Table 1. The threshold for ROR was the lower limit of the 95% CI > 1 and a ≥ 3. The threshold for PRR was PRR ≥ 2, χ^2^ ≥ 4, and a ≥ 3. The threshold for BCPNN was IC025 > 0. The threshold for MGPS was EBGM05 > 2. The specific calculation formulas and threshold criteria for each algorithm are provided in Supplementary Table 2.

#### 2.3.2 Time to onset (TTO) and weibull distribution analysis of AEs

Time to onset of Symdeko-related AEs was defined as the time interval between the AE onset date in the DEMO file and the drug start date in the THER file. For analysis convenience, all TTOs were consistently converted into days. Additionally, the Weibull distribution was further applied to assess the changes in the occurrence of AEs over time. In summary, we use the median and quartiles to describe the TTO, and employ the Weibull distribution to predict changes in the occurrence of AEs over time.

#### 2.3.3 Sensitivity analysis

Symdeko is commonly used in combination with drugs such as tiotropium bromide, pancreatic enzymes, prednisone, aztreonam, and dornase alfa. To more accurately reflect the AEs caused by Symdeko alone, this study further excluded reports of Symdeko in combination with these drugs and reanalyzed the data. This exclusion method effectively isolates the independent effects of Symdeko, thereby improving the accuracy of signal detection and the reliability of the study results. All data processing and statistical analyses were performed using R (Version 4.4.1).

## 3 Results

### 3.1 Descriptive analysis

During the monitoring period from Q1 2018 to Q3 2024, a total of 11,739,753 reports were submitted to the FAERS database. After strict screening and analysis, 5,245 AE reports related to Symdeko were analyzed. Among the Symdeko-related AE reports, 54.9% were female, 41.3% were male, and the remaining 3.8% had missing sex. In terms of age distribution, the 18–65 age group accounted for the largest proportion, reaching 40.0%. Of all Symdeko-related AE reports, 62.3% were submitted by healthcare professionals, 37.5% by non-healthcare professionals, and 0.2% had missing reporter information. Geographically, the highest proportion of reports came from the United States (90.2%), followed by the United Kingdom (7.1%), Australia (0.5%), Germany (0.4%), and Spain (0.4%). Detailed information on AE reports is provided in Table 1.

**TABLE 1:** Clinical characteristics of adverse event (AE) reports for Symdeko from the Food and Drug Administration Adverse Event Reporting System (FAERS) database (Q1 2018–Q3 2024).

Characteristics	Number of cases	Proportion of cases (%)
Numbers	5,245	–
**Gender**		
Male	2,167	41.3
Female	2,877	54.9
Missing	201	3.8
**Age**		
Median (IQR)	23 (17.33)	–
< 18	795	15.2
18–65	2,098	40.0
65–85	36	0.7
Missing	2,316	44.2
**Top 5 reported countries**		
United States	4,730	90.2
United Kingdom	371	7.1
Australia	25	0.5
Germany	22	0.4
Spain	22	0.4
**Reporter**		
Non-healthcare professional	1,966	37.5
Healthcare professional	3,268	62.3
Missing	11	0.2
**Reporting year**		
2018	1,039	19.8
2019	2,709	51.6
2020	1,099	21.0
2021	208	4.0
2022	79	1.5
2023	66	1.3
2024	45	0.9

IQR, interquartile range.

### 3.2 Distribution of AEs at the SOC level

**FIGURE 2:**
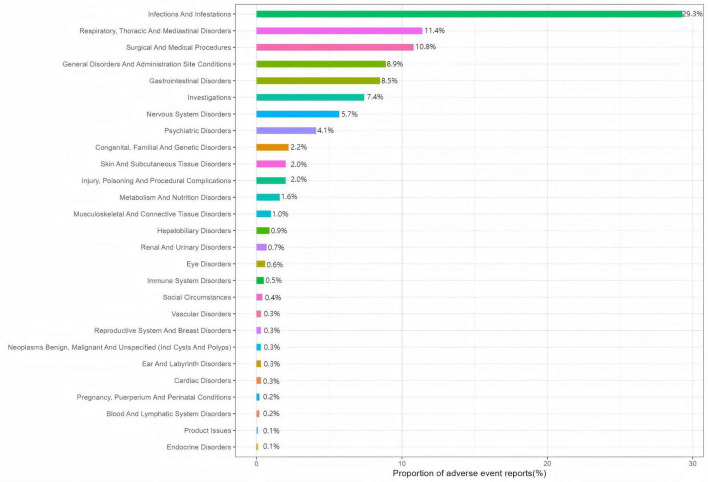
Adverse event (AE) distribution across system organ classes for Symdeko.

Symdeko-related AEs were distributed across 27 SOCs, with specific signal strengths shown in Table 2. At the SOC level, AEs meeting the positive criteria include: investigations, infections and infestations, congenital, familial and genetic disorders, respiratory, thoracic and mediastinal disorders, surgical and medical procedures. Figure 2 visually presents the distribution of AEs at the SOC level.

**TABLE 2:** Signal strength of Symdeko-associated adverse events (AEs) across system organ classes (SOC) in the Food and Drug Administration Adverse Event Reporting System (FAERS) database.

SOC	Case numbers	ROR (95% CI)	PRR (χ^2^)	EBGM (EBGM05)	IC (IC025)
Infections and infestations*	2,580	7.07 (6.75–7.4)	5.29 (9487.73)	5.28 (5.08)	2.4 (2.34)
Respiratory, thoracic and mediastinal disorders*	1,006	2.7 (2.52–2.88)	2.5 (949.32)	2.5 (2.37)	1.32 (1.23)
Surgical and medical procedures*	950	8.29 (7.75–8.87)	7.5 (5418.34)	7.49 (7.08)	2.9 (2.81)
General disorders and administration site conditions	782	0.46 (0.42–0.49)	0.5 (462.43)	0.5 (0.47)	−0.99 (−1.09)
Gastrointestinal disorders	747	1.06 (0.99–1.15)	1.06 (2.51)	1.06 (0.99)	0.08 (−0.03)
Investigations*	652	1.3 (1.2–1.41)	1.28 (42.17)	1.28 (1.2)	0.36 (0.24)
Nervous system disorders	499	0.74 (0.68–0.81)	0.75 (42.99)	0.75 (0.7)	−0.41 (−0.54)
Psychiatric disorders	364	0.77 (0.69–0.86)	0.78 (23.77)	0.78 (0.71)	−0.36 (−0.51)
Congenital, familial and genetic disorders*	191	8.13 (7.04–9.38)	7.97 (1165.31)	7.96 (7.06)	2.99 (2.78)
Injury, poisoning and procedural complications	178	0.15 (0.13–0.18)	0.17 (815.76)	0.17 (0.15)	−2.55 (−2.77)
Skin and subcutaneous tissue disorders	175	0.32 (0.28–0.37)	0.34 (243.79)	0.34 (0.3)	−1.57 (−1.79)
Metabolism and nutrition disorders	139	0.8 (0.68–0.95)	0.8 (6.86)	0.8 (0.7)	−0.32 (−0.56)
Musculoskeletal and connective tissue disorders	88	0.19 (0.15–0.23)	0.2 (304.16)	0.2 (0.17)	−2.35 (−2.65)
Hepatobiliary disorders	77	1.04 (0.83–1.3)	1.04 (0.11)	1.04 (0.86)	0.05 (−0.27)
Renal and urinary disorders	61	0.34 (0.26–0.43)	0.34 (79.66)	0.34 (0.28)	−1.56 (−1.92)
Eye disorders	52	0.3 (0.23–0.39)	0.3 (84.23)	0.3 (0.24)	−1.72 (−2.11)
Immune system disorders	42	0.38 (0.28–0.52)	0.39 (41.33)	0.39 (0.3)	−1.37 (−1.81)
Social circumstances	34	0.81 (0.58–1.14)	0.81 (1.46)	0.81 (0.61)	−0.3 (−0.79)
Vascular disorders	30	0.18 (0.12–0.26)	0.18 (112.78)	0.18 (0.13)	−2.46 (−2.98)
Neoplasms benign, malignant and unspecified (incl cysts and polyps)	27	0.09 (0.06–0.13)	0.09 (253.1)	0.09 (0.07)	−3.45 (−4)
Ear and labyrinth disorders	26	0.7 (0.48–1.04)	0.71 (3.2)	0.71 (0.51)	−0.5 (−1.06)
Cardiac disorders	26	0.14 (0.1–0.21)	0.15 (131.95)	0.15 (0.11)	−2.77 (−3.33)
Reproductive system and breast disorders	23	0.41 (0.27–0.62)	0.41 (19.24)	0.41 (0.29)	−1.27 (−1.86)
Blood and lymphatic system disorders	17	0.11 (0.07–0.18)	0.11 (119.83)	0.11 (0.08)	−3.14 (−3.82)
Pregnancy, puerperium and perinatal conditions	16	0.49 (0.3–0.79)	0.49 (8.66)	0.49 (0.32)	−1.04 (−1.74)
Product issues	7	0.04 (0.02–0.09)	0.04 (150.66)	0.04 (0.02)	−4.53 (−5.55)
Endocrine disorders	5	0.22 (0.09–0.52)	0.22 (14.29)	0.22 (0.1)	−2.21 (−3.39)

Asterisks (*) indicate statistically significant signals meeting four algorithm; ROR, reporting odds ratio; PRR, proportional reporting ratio; EBGM, empirical Bayesian geometric mean; EBGM05, the lower limit of the 95% CI of EBGM; IC, information component; IC025, the lower limit of the 95% CI of the IC; CI, confidence interval; PT, preferred term.

### 3.3 Distribution of AEs at the PT level

This study identified 276 positive signals, with Table 3 showing the top 50 AEs ranked by frequency among the positive signals. Common AEs such as headache, nausea, cough, nasal congestion, elevated liver enzymes, and abdominal pain were consistent with known AEs. Notably, this study discovered some novel signals, including anxiety, depression, suicidal ideation and renal stones. Additionally, all AEs that met the positive signal criteria are listed in Supplementary Table 3 (*N* > 5).

**TABLE 3:** Top 50 positive adverse events (AEs) of Symdeko at the PT level.

PT	Case numbers	ROR (95% CI)	PRR (χ^2^)	EBGM (EBGM05)	IC (IC025)
Infective pulmonary exacerbation of cystic fibrosis	1,197	1766.92 (1644.22–1898.78)	1526.55 (1253072.05)	1048.39 (987.12)	10.03 (9.93)
Hospitalization	572	24.2 (22.22–26.34)	22.69 (11812.06)	22.54 (20.99)	4.49 (4.37)
Infection	319	15.37 (13.74–17.19)	14.85 (4111.49)	14.79 (13.46)	3.89 (3.72)
Pneumonia	241	5.45 (4.79–6.19)	5.32 (849.52)	5.32 (4.78)	2.41 (2.22)
Headache	213	2.57 (2.25–2.95)	2.54 (199.86)	2.53 (2.26)	1.34 (1.14)
Cough	174	4.24 (3.65–4.93)	4.18 (421.89)	4.17 (3.68)	2.06 (1.84)
Cystic fibrosis	170	170.62 (146.05–199.32)	167.34 (26772.66)	159.41 (139.97)	7.32 (7.09)
Pulmonary function test decreased	149	229 (193.74–270.69)	225.14 (31153.81)	211 (183.45)	7.72 (7.48)
Cystic fibrosis respiratory infection suppression	149	945.55 (787.63–1135.14)	929.55 (108145.22)	727.57 (624.41)	9.51 (9.25)
Nausea	138	1.35 (1.14–1.59)	1.34 (12.11)	1.34 (1.16)	0.42 (0.18)
Hemoptysis	134	37.84 (31.88–44.92)	37.28 (4,681)	36.88 (31.95)	5.2 (4.95)
Dyspnea	130	1.71 (1.44–2.03)	1.7 (37.69)	1.7 (1.47)	0.76 (0.51)
Malaise	127	2.17 (1.82–2.59)	2.15 (78.97)	2.15 (1.86)	1.11 (0.85)
Nasopharyngitis	99	3.66 (3–4.46)	3.63 (188.91)	3.63 (3.07)	1.86 (1.57)
Influenza	84	5 (4.03–6.2)	4.96 (265.87)	4.96 (4.14)	2.31 (1.99)
Dizziness	82	1.3 (1.04–1.61)	1.29 (5.54)	1.29 (1.08)	0.37 (0.05)
Abdominal pain upper	81	2.98 (2.39–3.71)	2.96 (105.29)	2.96 (2.46)	1.56 (1.24)
Weight decreased	79	2.03 (1.63–2.53)	2.02 (40.81)	2.02 (1.68)	1.01 (0.69)
Sinusitis	73	5.07 (4.03–6.39)	5.04 (236.25)	5.03 (4.15)	2.33 (1.99)
Decreased appetite	65	1.96 (1.53–2.5)	1.95 (30.16)	1.95 (1.59)	0.96 (0.61)
Constipation	65	2.14 (1.68–2.73)	2.13 (39.28)	2.13 (1.74)	1.09 (0.74)
Chest discomfort	60	4.4 (3.42–5.68)	4.38 (156.63)	4.38 (3.54)	2.13 (1.76)
Anxiety	56	1.47 (1.13–1.91)	1.47 (8.42)	1.47 (1.18)	0.55 (0.17)
Depression	56	2.15 (1.65–2.8)	2.14 (34.18)	2.14 (1.72)	1.1 (0.71)
Lung transplant	52	90.11 (68.36–118.78)	89.58 (4436.38)	87.27 (69.26)	6.45 (6.04)
Productive cough	52	6.96 (5.3–9.14)	6.92 (263.17)	6.91 (5.5)	2.79 (2.39)
Abdominal pain	51	1.71 (1.3–2.25)	1.7 (14.85)	1.7 (1.35)	0.77 (0.37)
Liver function test increased	49	11.57 (8.74–15.33)	11.52 (469.15)	11.48 (9.07)	3.52 (3.11)
Forced expiratory volume decreased	43	80.67 (59.57–109.24)	80.28 (3287.97)	78.42 (60.85)	6.29 (5.85)
Lower respiratory tract infection	43	6.01 (4.45–8.11)	5.98 (178.29)	5.97 (4.65)	2.58 (2.14)
Pseudomonas infection	41	35.27 (25.91–48.01)	35.11 (1344.68)	34.75 (26.85)	5.12 (4.67)
Viral infection	41	9.33 (6.86–12.69)	9.29 (302.76)	9.27 (7.17)	3.21 (2.77)
Treatment non-compliance	39	5.28 (3.86–7.24)	5.26 (134.58)	5.26 (4.04)	2.39 (1.94)
Hepatic enzyme increased	38	3.88 (2.82–5.34)	3.87 (80.87)	3.87 (2.96)	1.95 (1.49)
Sinus operation	37	111.33 (80.18–154.57)	110.86 (3899.16)	107.34 (81.56)	6.75 (6.27)
General physical health deterioration	37	2.25 (1.63–3.1)	2.24 (25.47)	2.24 (1.71)	1.16 (0.69)
Gastrointestinal disorder	36	2.72 (1.96–3.77)	2.71 (38.92)	2.71 (2.06)	1.44 (0.96)
Migraine	35	2.53 (1.82–3.53)	2.53 (32.3)	2.52 (1.91)	1.34 (0.85)
Respiratory symptom	33	60.26 (42.68–85.08)	60.04 (1882.1)	59 (44.21)	5.88 (5.38)
Alanine aminotransferase increased	28	4.09 (2.82–5.93)	4.08 (65.02)	4.07 (2.99)	2.03 (1.49)
Nephrolithiasis	28	4.23 (2.91–6.12)	4.21 (68.62)	4.21 (3.09)	2.07 (1.54)
Aspartate aminotransferase increased	27	4.79 (3.28–7)	4.78 (80.68)	4.78 (3.48)	2.26 (1.71)
Oropharyngeal pain	27	2.02 (1.38–2.94)	2.01 (13.8)	2.01 (1.47)	1.01 (0.46)
Lung disorder	27	3.89 (2.66–5.67)	3.88 (57.67)	3.88 (2.82)	1.95 (1.41)
Respiratory tract infection	26	6.77 (4.61 - 9.95)	6.75 (127.26)	6.74 (4.88)	2.75 (2.2)
Intestinal obstruction	26	5.03 (3.42–7.39)	5.02 (83.51)	5.01 (3.63)	2.32 (1.77)
Dyspepsia	25	2.06 (1.39–3.04)	2.05 (13.51)	2.05 (1.48)	1.04 (0.47)
Respiratory tract congestion	25	12.82 (8.65–18.99)	12.78 (270.59)	12.74 (9.17)	3.67 (3.1)
Suicidal ideation	23	2.21 (1.47–3.33)	2.21 (15.26)	2.21 (1.57)	1.14 (0.55)
Sinus disorder	22	7.74 (5.09–11.77)	7.73 (128.58)	7.71 (5.43)	2.95 (2.34)

ROR, reporting odds ratio; PRR, proportional reporting ratio; EBGM, empirical Bayesian geometric mean; EBGM05, the lower limit of the 95% CI of EBGM; IC, information component; IC025, the lower limit of the 95% CI of the IC; CI, confidence interval; PT, preferred term.

### 3.4 Subgroup analysis

Gender subgroup analysis revealed that among the 30 most frequently reported positive AEs, dizziness, anxiety, and lower respiratory infections were exclusively observed in females, whereas nausea and abdominal pain were solely reported in males. Detailed information can be found in Supplementary Tables 4, 5. Age subgroup analysis indicated that among the 30 most commonly reported positive AEs, decreased appetite and intestinal obstruction were uniquely observed in individuals under 18, whereas kidney stones occurred exclusively in those aged 18 and older. Specific details are available in Supplementary Tables 6, 7.

### 3.5 TTO and weibull distribution analysis of Symdeko-related AEs

**FIGURE 3:**
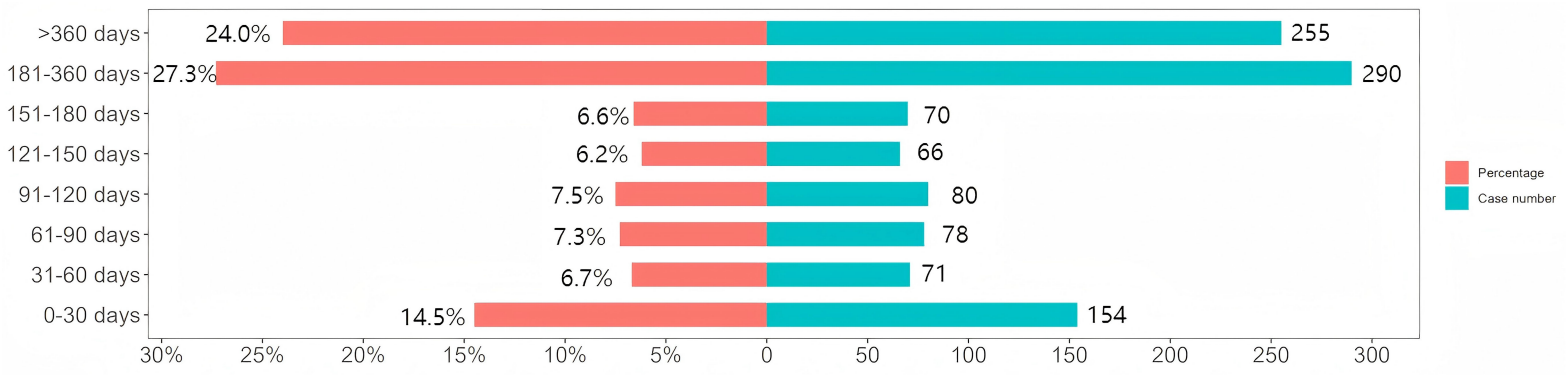
Time to onset of adverse events (AEs) associated with Symdeko.

We analyzed 1,064 reports providing the TTO of AEs. The median TTO of AEs was 188 days [interquartile range (IQR): 78–353 days]. As shown in Figure 3, the majority of AEs occurred within 6–12 months (*n* = 290, 27.3%) and over 12 months (*n* = 255, 24.0%) after the initial treatment. Notably, the incidence of AEs within the first month after initial treatment was 14.5%. Weibull distribution analysis showed that the TTO of AEs followed a wear-out failure model, with a shape parameter (β) of 1.10 [95% confidence interval (CI): 1.04–1.15]. The remaining parameters are shown in Table 4.

**TABLE 4:** Time to onset of Symdeko-associated adverse events (AEs) and weibull distribution analysis.

Drug	TTO (days)		Weibull distribution		
	Case reports	Median (d) (IQR)	Scale parameter: α (95% CI)	Shape parameter: β (95% CI)	Type
Symdeko	1,064	188 (78–353)	232.6 (219.2–245.9)	1.10 (1.04–1.15)	Wear-out failure

TTO, time to onset; CI, confidence interval; IQR, interquartile range.

### 3.6 Sensitivity analysis

To enhance the reliability of the results, sensitivity analysis was performed. Symdeko is commonly co-administered with tiotropium bromide, pancreatic enzymes, prednisone, aztreonam, and dornase alfa. After excluding reports involving Symdeko co-administered with these drugs, the analysis was re-performed. The positive AEs observed in the sensitivity analysis were largely consistent with the initial findings, with headache, nausea, anxiety, depression, suicidal ideation, and renal stones still showing positive signals (Supplementary Table 8).

## 4 Discussion

This study evaluated the real world safety of Symdeko using the FAERS database. We confirmed adverse events listed on the label, including headache, nausea, and elevated liver enzymes. We also identified novel signals such as anxiety, depression, suicidal ideation, and renal stones. These findings provide initial evidence to support safer and more informed use of Symdeko in practice.

Headache is a well-known AE listed on the drug label. In a phase III clinical trial of Symdeko for the treatment of CF, the incidence of headache was 14.8% (22). In another long-term clinical trial of Symdeko for CF, headache remained a common AE (23). These findings are consistent with the results of this study. The occurrence of headache may be related to CFTR’s effects on the nervous system, vascular dilation and constriction, or the modulation of neurotransmitters, which may influence neuronal activity, cerebral blood vessel expansion or contraction, and norepinephrine levels, thereby triggering headaches. Headache can significantly disrupt patients’ daily lives, work, and studies, potentially affecting medication adherence (24). Therefore, we recommend that clinicians inform patients about the risk of headache before initiating treatment and conduct continuous monitoring. If pain occurs, clinicians should consider the principle of graded analgesia and promptly alleviate the patient’s symptoms to avoid the risk of premature discontinuation of the medication.

Nausea is another common known AE, and multiple studies have also indicated that nausea is a frequent AE (12, 25, 26), consistent with the findings of this study. It is noteworthy that a systematic review on the real-world safety of CFTR modulator therapy for CF indicates that in some studies, persistent nausea may lead to discontinuation of the treatment (27). Persistent nausea can lead to vomiting, potentially resulting in electrolyte imbalances. Given that CF is a disorder of the exocrine glands, these electrolyte imbalances may exacerbate the characteristic clinical symptoms of CF, such as increased coughing, sputum production, and dyspnea. Furthermore, chronic nausea may impair nutrient absorption, which has been linked to increased mortality and decreased pulmonary function in patients (28). Therefore, nausea symptoms following Symdeko use warrant close monitoring to prevent further complications or exacerbation of pre-existing conditions. If a patient experiences persistent nausea accompanied by vomiting, clinicians should promptly administer antiemetics and provide appropriate fluid supplementation.

Additionally, this study identified several novel signals, including anxiety, depression, suicidal ideation and renal stones. Psychiatric adverse events have been more commonly reported with other CFTR modulators, such as Trikafta. An observational study showed that PHQ-9 and GAD-7 scores increased after Trikafta administration (29). Multiple case reports have suggested that Trikafta may induce anxiety, depression, and suicidal ideation (30, 31). Researchers have proposed a potential mechanism: the rostral anterior cingulate cortex (rACC) plays a key role in the neural circuitry of emotion regulation, and Trikafta may directly modulate 5-hydroxytryptamine 2A (5-HT2A) receptors in the rACC and influence gamma-aminobutyric acid (GABA) function by regulating chloride ion levels (32). This study found a statistical association between Symdeko and psychiatric AEs. Furthermore, considering the similarity in mechanisms between Symdeko and Trikafta, as well as the significant impact of psychiatric AEs on quality of life, clinicians may need to assess the psychological health of CF patients before starting Symdeko and continue monitoring them during treatment to prevent severe AEs such as suicide. Based on this, we recommend psychological interventions, such as peer support and motivational interviewing, which may help manage various mental health issues and alleviate the emotional burden of CF patients (33)

Moreover, this study identified renal stones as another novel signal associated with Symdeko, which met the positive threshold for all four disproportionality analyses. Renal stones primarily manifest as flank pain and, in severe cases, may be accompanied by nausea and vomiting (34). Special attention should be given to CF patients developing renal stones, as the use of opioid analgesics may lead to respiratory depression, exacerbating respiratory symptoms. Additionally, renal stones in CF patients appear to be prone to recurrence (35). The relationship between Symdeko and renal stone formation, as well as its potential mechanisms, remains unclear and may be influenced by multiple factors. First, magnesium metabolism may play a role. Recent studies have shown that patients receiving Trikafta, a CFTR modulator, exhibited significantly reduced urinary magnesium levels, which is a known risk factor for urolithiasis (36, 37). Since Symdeko contains key components of Trikafta, whether it induces similar metabolic changes warrants further investigation. Additionally, renal stone formation may be associated with factors beyond Symdeko itself. CF patients carrying CFTR gene mutations often experience pancreatic exocrine insufficiency and impaired fatty acid absorption, leading to abnormally high levels of fatty acids in the intestine. Fatty acids bind to calcium, reducing the availability of free calcium to complex with oxalate. As a result, unbound oxalate is absorbed in the intestine, leading to hyperoxaluria and the formation of calcium oxalate stones (38, 39). Finally, long-term and frequent antibiotic use may disrupt the gut microbiota, resulting in excessive endogenous oxalate absorption and the subsequent formation of hyperoxaluria-related stones (39–41). Therefore, a sensitivity analysis was conducted in this study, excluding reports involving Symdeko co-administered with commonly used antibiotics, followed by a reanalysis of disproportionality signals, where renal stones continued to exhibit a positive signal. In conclusion, a potential association may exist between Symdeko and renal stone formation, which requires validation through future prospective studies. Furthermore, renal stones can significantly disrupt family and social life, occupational activities, personal finances, and emotional and psychological wellbeing. Thus, healthcare professionals should monitor the occurrence of renal stones during Symdeko treatment and intervene when necessary.

Gender-related subgroup analysis indicated that dizziness and anxiety require particular attention in females, while nausea and abdominal pain should be noted in males. In individuals under 18 years of age, the occurrence of decreased appetite and intestinal obstruction should be monitored, whereas kidney stones should be a focus in patients aged 18 years and older. These findings provide new insights into the clinical use of Symdeko in different gender and age groups, but further investigation is required to elucidate the underlying mechanisms.

The TTO analysis showed that the median onset time for Symdeko-related AEs was 188 days (IQR: 78–353 days). The majority of AEs occurred more than six months after treatment initiation (*n* = 545, 51.3%). Notably, a considerable number of AEs were also observed within the first six months of treatment, with the highest incidence occurring in the first month (*n* = 154, 14.5%). Additionally, the Weibull distribution was used to model the occurrence of AEs over time, with results aligning with the wear-out failure model, indicating that the likelihood of drug-induced AEs increases as time progresses. These findings highlight the necessity of heightened vigilance during the first month of treatment and emphasize the importance of long-term monitoring for patients receiving Symdeko.

After excluding reports involving the co-administration of Symdeko with tiotropium bromide, pancreatic enzymes, prednisone, aztreonam, and dornase alfa, a disproportionality analysis was re-conducted. AEs such as headache, nausea, anxiety, depression, suicidal ideation, and renal stones continued to exhibit positive signals. These AEs warrant attention, as they may lead to a decline in patients’ quality of life and adherence, potentially impacting the therapeutic efficacy of the drug.

However, this study has several limitations. First, the FAERS database is based on voluntary reports from clinicians, pharmacists, nurses, and consumers, which may introduce reporting bias (15). Second, underreporting, missing data, and misreporting of AEs may affect the accuracy of the results. Additionally, the majority of reports originate from the United States, potentially limiting the external validity of the findings, highlighting the need for future studies incorporating AE reports from a broader range of regions. Another limitation of this study is that an AE was considered a positive signal if it met the threshold of any single disproportionality method, which may increase the false positive rate and therefore warrants more cautious interpretation of the results. Moreover, although sensitivity analysis was conducted to exclude the influence of commonly used medications, other confounding factors may still exist, necessitating cautious interpretation of the results. Finally, disproportionality analysis only reflects a statistical association between Symdeko and AEs, rather than a causal relationship (17). Large-scale prospective studies are needed to validate these findings in the future.

## 5 Conclusion

This study conducted a comprehensive evaluation of the real-world safety of Symdeko using the FAERS database. The study confirmed several known AEs, such as headache, nausea, and elevated liver enzymes, and identified some novel signals, including anxiety, depression, suicidal tendencies, and renal stones. The cumulative incidence of AEs after 6 months of treatment was 51.3%, indicating the need for ongoing monitoring during therapy. These findings provide preliminary real-world safety insights into Symdeko, aiding clinicians in safer drug utilization. Future large-scale prospective studies are necessary to further validate the findings of this study.

## Data Availability

The datasets presented in this study can be found in online repositories. The names of the repository/repositories and accession number(s) can be found in the article/Supplementary material.
