# Supplementary material for: Real-world safety of Symdeko: insights from the food and drug administration adverse event reporting system
Source: Front Med (Lausanne). 2025 Oct 30;12:1681985. doi: 10.3389/fmed.2025.1681985 (PMC12611701; doi:10.3389/fmed.2025.1681985)
Supplement: Supplementary file 1 [file Table_1.docx]

Supplementary Material

Supplementary Tables

Supplementary Table 1:

Two-by-two contingency table for disproportionality analyses.

|  | Target AEs | Other AEs | Total |
| --- | --- | --- | --- |
| Symdeko | a | b | a+b |
| Other drugs | c | d | c+d |
| Total | a+c | b+d | a+b+c+d |

Abbreviation: AEs, adverse events; a, number of reports containing both the target drug and target adverse drug reaction; b, number of reports containing other adverse drug reaction of the target drug; c, number of reports containing the target adverse drug reaction of other drugs; d, number of reports containing other drugs and other adverse drug reactions.

**Supplementary Table 2**:

Four major algorithms used for signal detection.

| Algorithms | Equation | Criteria |
| --- | --- | --- |
| ROR | ROR=ad/b/c | lower limit of 95% CI>1, N≥3 |
|  | 95%CI=e^ln(ROR)±1.96(1/a+1/b+1/c+1/d)^0.5^ |  |
| PRR | PRR=a(c+d)/c/(a+b) | PRR≥2, χ^2^≥4, N≥3 |
|  | χ^2^=[(ad-bc)^2](a+b+c+d)/[(a+b)(c+d)(a+c)(b+d)] |  |
| BCPNN | IC=log_2_a(a+b+c+d)(a+c)(a+b) | IC025>0 |
|  | 95%CI= E(IC) ± 2V(IC)^0.5 |  |
| MGPS | EBGM=a(a+b+c+d)/(a+c)/(a+b) | EBGM05>2 |
|  | 95%CI=e^ln(EBGM)±1.96(1/a+1/b+1/c+1/d)^0.5^ |  |

Abbreviation: a, number of reports containing both the target drug and target adverse drug reaction; b, number of reports containing other adverse drug reaction of the target drug; c, number of reports containing the target adverse drug reaction of other drugs; d, number of reports containing other drugs and other adverse drug reactions. 95%CI, 95% confidence interval; N, the number of reports; χ2, chi-squared; IC, information component; IC025, the lower limit of 95% CI of the IC; E(IC), the IC expectations; V(IC), the variance of IC; EBGM, empirical Bayesian geometric mean; EBGM05, the lower limit of 95% CI of EBGM.

**Supplementary Table 3**:

All adverse events meeting the positive signal threshold at the PT level（Case number（N）＞5）

| PT | Case numbers | ROR(95%CI) | PRR(χ^2^) | EBGM(EBGM05) | IC(IC025) |
| --- | --- | --- | --- | --- | --- |
| Infective Pulmonary Exacerbation Of Cystic Fibrosis | 1197 | 1766.92(1644.22-1898.78 ) | 1526.55(1253072.05) | 1048.39( 987.12 ) | 10.03 ( 9.93 ) |
| Hospitalisation | 572 | 24.2(22.22- 26.34 ) | 22.69 ( 11812.06 ) | 22.54 ( 20.99 ) | 4.49 ( 4.37 ) |
| Infection | 319 | 15.37(13.74-17.19) | 14.85 ( 4111.49 ) | 14.79 ( 13.46 ) | 3.89 ( 3.72 ) |
| Pneumonia | 241 | 5.45 ( 4.79 - 6.19 ) | 5.32 ( 849.52 ) | 5.32 ( 4.78 ) | 2.41 ( 2.22 ) |
| Headache | 213 | 2.57 ( 2.25 - 2.95 ) | 2.54 ( 199.86 ) | 2.53 ( 2.26 ) | 1.34 ( 1.14 ) |
| Cough | 174 | 4.24 ( 3.65 - 4.93 ) | 4.18 ( 421.89 ) | 4.17 ( 3.68 ) | 2.06 ( 1.84 ) |
| Cystic Fibrosis | 170 | 170.62(146.05-199.32) | 167.34(26772.66 ) | 159.41 ( 139.97 ) | 7.32 ( 7.09 ) |
| Pulmonary Function Test Decreased | 149 | 229(193.74-270.69) | 225.14(31153.81 ) | 211 ( 183.45 ) | 7.72 ( 7.48 ) |
| Cystic Fibrosis Respiratory Infection Suppression | 149 | 945.55(787.63-1135.14 ) | 929.55(108145.22 ) | 727.57 ( 624.41 ) | 9.51 ( 9.25 ) |
| Nausea | 138 | 1.35 ( 1.14 - 1.59 ) | 1.34 ( 12.11 ) | 1.34 ( 1.16 ) | 0.42 ( 0.18 ) |
| Haemoptysis | 134 | 37.84 ( 31.88 - 44.92 ) | 37.28 ( 4681 ) | 36.88 ( 31.95 ) | 5.2 ( 4.95 ) |
| Dyspnoea | 130 | 1.71 ( 1.44 - 2.03 ) | 1.7 ( 37.69 ) | 1.7 ( 1.47 ) | 0.76 ( 0.51 ) |
| Malaise | 127 | 2.17 ( 1.82 - 2.59 ) | 2.15 ( 78.97 ) | 2.15 ( 1.86 ) | 1.11 ( 0.85 ) |
| Nasopharyngitis | 99 | 3.66 ( 3 - 4.46 ) | 3.63 ( 188.91 ) | 3.63 ( 3.07 ) | 1.86 ( 1.57 ) |
| Influenza | 84 | 5 ( 4.03 - 6.2 ) | 4.96 ( 265.87 ) | 4.96 ( 4.14 ) | 2.31 ( 1.99 ) |
| Dizziness | 82 | 1.3 ( 1.04 - 1.61 ) | 1.29 ( 5.54 ) | 1.29 ( 1.08 ) | 0.37 ( 0.05 ) |
| Abdominal Pain Upper | 81 | 2.98 ( 2.39 - 3.71 ) | 2.96 ( 105.29 ) | 2.96 ( 2.46 ) | 1.56 ( 1.24 ) |
| Weight Decreased | 79 | 2.03 ( 1.63 - 2.53 ) | 2.02 ( 40.81 ) | 2.02 ( 1.68 ) | 1.01 ( 0.69 ) |
| Sinusitis | 73 | 5.07 ( 4.03 - 6.39 ) | 5.04 ( 236.25 ) | 5.03 ( 4.15 ) | 2.33 ( 1.99 ) |
| Decreased Appetite | 65 | 1.96 ( 1.53 - 2.5 ) | 1.95 ( 30.16 ) | 1.95 ( 1.59 ) | 0.96 ( 0.61 ) |
| Constipation | 65 | 2.14 ( 1.68 - 2.73 ) | 2.13 ( 39.28 ) | 2.13 ( 1.74 ) | 1.09 ( 0.74 ) |
| Chest Discomfort | 60 | 4.4 ( 3.42 - 5.68 ) | 4.38 ( 156.63 ) | 4.38 ( 3.54 ) | 2.13 ( 1.76 ) |
| Anxiety | 56 | 1.47 ( 1.13 - 1.91 ) | 1.47 ( 8.42 ) | 1.47 ( 1.18 ) | 0.55 ( 0.17 ) |
| Depression | 56 | 2.15 ( 1.65 - 2.8 ) | 2.14 ( 34.18 ) | 2.14 ( 1.72 ) | 1.1 ( 0.71 ) |
| Lung Transplant | 52 | 90.11 ( 68.36 - 118.78 ) | 89.58 ( 4436.38 ) | 87.27 ( 69.26 ) | 6.45 ( 6.04 ) |
| Productive Cough | 52 | 6.96 ( 5.3 - 9.14 ) | 6.92 ( 263.17 ) | 6.91 ( 5.5 ) | 2.79 ( 2.39 ) |
| Abdominal Pain | 51 | 1.71 ( 1.3 - 2.25 ) | 1.7 ( 14.85 ) | 1.7 ( 1.35 ) | 0.77 ( 0.37 ) |
| Liver Function Test Increased | 49 | 11.57 ( 8.74 - 15.33 ) | 11.52 ( 469.15 ) | 11.48 ( 9.07 ) | 3.52 ( 3.11 ) |
| Forced Expiratory Volume Decreased | 43 | 80.67 ( 59.57 - 109.24 ) | 80.28 ( 3287.97 ) | 78.42 ( 60.85 ) | 6.29 ( 5.85 ) |
| Lower Respiratory Tract Infection | 43 | 6.01 ( 4.45 - 8.11 ) | 5.98 ( 178.29 ) | 5.97 ( 4.65 ) | 2.58 ( 2.14 ) |
| Pseudomonas Infection | 41 | 35.27 ( 25.91 - 48.01 ) | 35.11 ( 1344.68 ) | 34.75 ( 26.85 ) | 5.12 ( 4.67 ) |
| Viral Infection | 41 | 9.33 ( 6.86 - 12.69 ) | 9.29 ( 302.76 ) | 9.27 ( 7.17 ) | 3.21 ( 2.77 ) |
| Treatment Noncompliance | 39 | 5.28 ( 3.86 - 7.24 ) | 5.26 ( 134.58 ) | 5.26 ( 4.04 ) | 2.39 ( 1.94 ) |
| Hepatic Enzyme Increased | 38 | 3.88 ( 2.82 - 5.34 ) | 3.87 ( 80.87 ) | 3.87 ( 2.96 ) | 1.95 ( 1.49 ) |
| Sinus Operation | 37 | 111.33 ( 80.18 - 154.57 ) | 110.86 ( 3899.16 ) | 107.34 ( 81.56 ) | 6.75 ( 6.27 ) |
| General Physical Health Deterioration | 37 | 2.25 ( 1.63 - 3.1 ) | 2.24 ( 25.47 ) | 2.24 ( 1.71 ) | 1.16 ( 0.69 ) |
| Gastrointestinal Disorder | 36 | 2.72 ( 1.96 - 3.77 ) | 2.71 ( 38.92 ) | 2.71 ( 2.06 ) | 1.44 ( 0.96 ) |
| Migraine | 35 | 2.53 ( 1.82 - 3.53 ) | 2.53 ( 32.3 ) | 2.52 ( 1.91 ) | 1.34 ( 0.85 ) |
| Respiratory Symptom | 33 | 60.26 ( 42.68 - 85.08 ) | 60.04 ( 1882.1 ) | 59 ( 44.21 ) | 5.88 ( 5.38 ) |
| Alanine Aminotransferase Increased | 28 | 4.09 ( 2.82 - 5.93 ) | 4.08 ( 65.02 ) | 4.07 ( 2.99 ) | 2.03 ( 1.49 ) |
| Nephrolithiasis | 28 | 4.23 ( 2.91 - 6.12 ) | 4.21 ( 68.62 ) | 4.21 ( 3.09 ) | 2.07 ( 1.54 ) |
| Aspartate Aminotransferase Increased | 27 | 4.79 ( 3.28 - 7 ) | 4.78 ( 80.68 ) | 4.78 ( 3.48 ) | 2.26 ( 1.71 ) |
| Oropharyngeal Pain | 27 | 2.02 ( 1.38 - 2.94 ) | 2.01 ( 13.8 ) | 2.01 ( 1.47 ) | 1.01 ( 0.46 ) |
| Lung Disorder | 27 | 3.89 ( 2.66 - 5.67 ) | 3.88 ( 57.67 ) | 3.88 ( 2.82 ) | 1.95 ( 1.41 ) |
| Respiratory Tract Infection | 26 | 6.77 ( 4.61 - 9.95 ) | 6.75 ( 127.26 ) | 6.74 ( 4.88 ) | 2.75 ( 2.2 ) |
| Intestinal Obstruction | 26 | 5.03 ( 3.42 - 7.39 ) | 5.02 ( 83.51 ) | 5.01 ( 3.63 ) | 2.32 ( 1.77 ) |
| Dyspepsia | 25 | 2.06 ( 1.39 - 3.04 ) | 2.05 ( 13.51 ) | 2.05 ( 1.48 ) | 1.04 ( 0.47 ) |
| Respiratory Tract Congestion | 25 | 12.82 ( 8.65 - 18.99 ) | 12.78 ( 270.59 ) | 12.74 ( 9.17 ) | 3.67 ( 3.1 ) |
| Suicidal Ideation | 23 | 2.21 ( 1.47 - 3.33 ) | 2.21 ( 15.26 ) | 2.21 ( 1.57 ) | 1.14 ( 0.55 ) |
| Sinus Disorder | 22 | 7.74 ( 5.09 - 11.77 ) | 7.73 ( 128.58 ) | 7.71 ( 5.43 ) | 2.95 ( 2.34 ) |
| Surgery | 22 | 2.72 ( 1.79 - 4.14 ) | 2.72 ( 23.93 ) | 2.72 ( 1.92 ) | 1.44 ( 0.84 ) |
| Pneumothorax | 22 | 9.84 ( 6.47 - 14.97 ) | 9.82 ( 173.85 ) | 9.8 ( 6.9 ) | 3.29 ( 2.69 ) |
| Upper Respiratory Tract Infection | 22 | 3.49 ( 2.3 - 5.3 ) | 3.48 ( 38.91 ) | 3.48 ( 2.45 ) | 1.8 ( 1.2 ) |
| Dysphonia | 21 | 2.58 ( 1.68 - 3.96 ) | 2.57 ( 20.21 ) | 2.57 ( 1.8 ) | 1.36 ( 0.75 ) |
| Abdominal Distension | 21 | 1.57 ( 1.02 - 2.41 ) | 1.57 ( 4.33 ) | 1.57 ( 1.1 ) | 0.65 ( 0.03 ) |
| Rhinovirus Infection | 21 | 30.18 ( 19.63 - 46.4 ) | 30.11 ( 585.82 ) | 29.85 ( 20.83 ) | 4.9 ( 4.28 ) |
| Staphylococcal Infection | 21 | 5.69 ( 3.7 - 8.73 ) | 5.67 ( 80.76 ) | 5.67 ( 3.96 ) | 2.5 ( 1.89 ) |
| Insurance Issue | 21 | 7.64 ( 4.98 - 11.73 ) | 7.62 ( 120.63 ) | 7.61 ( 5.32 ) | 2.93 ( 2.31 ) |
| Sinus Congestion | 20 | 12.16 ( 7.83 - 18.87 ) | 12.13 ( 203.57 ) | 12.09 ( 8.37 ) | 3.6 ( 2.96 ) |
| Respiratory Failure | 20 | 2.24 ( 1.44 - 3.47 ) | 2.23 ( 13.63 ) | 2.23 ( 1.55 ) | 1.16 ( 0.53 ) |
| Sputum Increased | 20 | 57.58 ( 36.99 - 89.64 ) | 57.45 ( 1090.75 ) | 56.5 ( 39.02 ) | 5.82 ( 5.18 ) |
| Nasal Congestion | 19 | 2.24 ( 1.43 - 3.52 ) | 2.24 ( 13.08 ) | 2.24 ( 1.54 ) | 1.16 ( 0.52 ) |
| Depressed Mood | 19 | 2.61 ( 1.67 - 4.1 ) | 2.61 ( 18.86 ) | 2.61 ( 1.79 ) | 1.38 ( 0.74 ) |
| Fungal Infection | 18 | 3.86 ( 2.43 - 6.13 ) | 3.85 ( 38.03 ) | 3.85 ( 2.61 ) | 1.95 ( 1.28 ) |
| Pulmonary Haemorrhage | 18 | 20.24 ( 12.73 - 32.18 ) | 20.2 ( 326.51 ) | 20.08 ( 13.62 ) | 4.33 ( 3.66 ) |
| Blood Alkaline Phosphatase Increased | 17 | 7.04 ( 4.37 - 11.33 ) | 7.02 ( 87.67 ) | 7.01 ( 4.71 ) | 2.81 ( 2.13 ) |
| Influenza Like Illness | 17 | 1.87 ( 1.16 - 3.02 ) | 1.87 ( 6.91 ) | 1.87 ( 1.26 ) | 0.9 ( 0.22 ) |
| Exposure During Pregnancy | 17 | 1.72 ( 1.07 - 2.77 ) | 1.72 ( 5.12 ) | 1.72 ( 1.15 ) | 0.78 ( 0.1 ) |
| Lethargy | 15 | 2.28 ( 1.37 - 3.78 ) | 2.28 ( 10.73 ) | 2.28 ( 1.49 ) | 1.19 ( 0.46 ) |
| Distal Intestinal Obstruction Syndrome | 15 | 249.97(147.87 - 422.57 ) | 249.54 ( 3455.37 ) | 232.28 ( 149.7 ) | 7.86 ( 7.11 ) |
| Liver Disorder | 14 | 2.42 ( 1.43 - 4.08 ) | 2.42 ( 11.61 ) | 2.41 ( 1.56 ) | 1.27 ( 0.53 ) |
| Emergency Care | 14 | 23.41 ( 13.83 - 39.61 ) | 23.37 ( 297.75 ) | 23.22 ( 14.95 ) | 4.54 ( 3.79 ) |
| Pneumonia Pseudomonal | 13 | 47.27 ( 27.33 - 81.75 ) | 47.2 ( 579.7 ) | 46.56 ( 29.44 ) | 5.54 ( 4.76 ) |
| Appendicectomy | 12 | 29.01 ( 16.43 - 51.23 ) | 28.97 ( 321.31 ) | 28.73 ( 17.85 ) | 4.84 ( 4.04 ) |
| Blood Glucose Decreased | 11 | 2.13 ( 1.18 - 3.85 ) | 2.13 ( 6.6 ) | 2.13 ( 1.3 ) | 1.09 ( 0.26 ) |
| Bronchial Secretion Retention | 11 | 50.31 ( 27.73 - 91.28 ) | 50.25 ( 523.12 ) | 49.52 ( 30.08 ) | 5.63 ( 4.79 ) |
| Bacterial Infection | 11 | 4.24 ( 2.35 - 7.67 ) | 4.24 ( 27.21 ) | 4.24 ( 2.58 ) | 2.08 ( 1.25 ) |
| Gamma-Glutamyltransferase Increased | 11 | 4.62 ( 2.56 - 8.35 ) | 4.62 ( 31.13 ) | 4.61 ( 2.81 ) | 2.21 ( 1.37 ) |
| Mental Disorder | 11 | 1.83 ( 1.01 - 3.3 ) | 1.82 ( 4.1 ) | 1.82 ( 1.11 ) | 0.87 ( 0.03 ) |
| Abortion Spontaneous | 11 | 2.22 ( 1.23 - 4.02 ) | 2.22 ( 7.38 ) | 2.22 ( 1.35 ) | 1.15 ( 0.32 ) |
| Sputum Discoloured | 10 | 6.11 ( 3.28 - 11.36 ) | 6.1 ( 42.59 ) | 6.09 ( 3.62 ) | 2.61 ( 1.74 ) |
| Mood Swings | 10 | 3.05 ( 1.64 - 5.68 ) | 3.05 ( 13.77 ) | 3.05 ( 1.81 ) | 1.61 ( 0.74 ) |
| Blood Bilirubin Increased | 9 | 3.12 ( 1.62 - 6 ) | 3.12 ( 12.95 ) | 3.12 ( 1.8 ) | 1.64 ( 0.73 ) |
| Increased Bronchial Secretion | 9 | 22.96 ( 11.92 - 44.24 ) | 22.94 ( 187.56 ) | 22.79 ( 13.16 ) | 4.51 ( 3.59 ) |
| Ear Infection | 9 | 2.23 ( 1.16 - 4.29 ) | 2.23 ( 6.11 ) | 2.23 ( 1.29 ) | 1.16 ( 0.24 ) |
| Pneumonia Bacterial | 9 | 6.08 ( 3.16 - 11.71 ) | 6.08 ( 38.14 ) | 6.07 ( 3.51 ) | 2.6 ( 1.69 ) |
| Mood Altered | 9 | 2.84 ( 1.48 - 5.46 ) | 2.84 ( 10.71 ) | 2.84 ( 1.64 ) | 1.5 ( 0.59 ) |
| Pharyngitis Streptococcal | 9 | 5.89 ( 3.06 - 11.33 ) | 5.89 ( 36.44 ) | 5.88 ( 3.4 ) | 2.56 ( 1.64 ) |
| Pneumonia Staphylococcal | 9 | 45.99 ( 23.82 - 88.82 ) | 45.95 ( 390.36 ) | 45.34 ( 26.14 ) | 5.5 ( 4.58 ) |
| Cystic Fibrosis Lung | 8 | 486.82(231.82-1022.34 ) | 486.38 ( 3383.01 ) | 424.75 ( 228.3 ) | 8.73 ( 7.7 ) |
| Blood Glucose Abnormal | 8 | 2.98 ( 1.49 - 5.96 ) | 2.98 ( 10.49 ) | 2.97 ( 1.66 ) | 1.57 ( 0.61 ) |
| Mycobacterium Avium Complex Infection | 8 | 19.85 ( 9.9 - 39.78 ) | 19.83 ( 142.21 ) | 19.72 ( 11.02 ) | 4.3 ( 3.34 ) |
| Hepatic Cirrhosis | 8 | 3.27 ( 1.63 - 6.54 ) | 3.27 ( 12.58 ) | 3.27 ( 1.83 ) | 1.71 ( 0.74 ) |
| Aspergillus Infection | 8 | 6.39 ( 3.19 - 12.79 ) | 6.38 ( 36.26 ) | 6.37 ( 3.57 ) | 2.67 ( 1.71 ) |
| Gastroenteritis Viral | 8 | 3.2 ( 1.6 - 6.4 ) | 3.2 ( 12.07 ) | 3.19 ( 1.79 ) | 1.68 ( 0.71 ) |
| Bronchopulmonary Aspergillosis Allergic | 8 | 43.32 ( 21.56 - 87.05 ) | 43.29 ( 326.26 ) | 42.75 ( 23.84 ) | 5.42 ( 4.45 ) |
| Cystic Fibrosis Related Diabetes | 8 | 322.59 ( 156.1 - 666.67 ) | 322.3 ( 2337.17 ) | 294.06 ( 160.19 ) | 8.2 ( 7.19 ) |
| Anger | 8 | 2.16 ( 1.08 - 4.32 ) | 2.16 ( 4.96 ) | 2.16 ( 1.21 ) | 1.11 ( 0.15 ) |
| Cholelithiasis | 7 | 2.16 ( 1.03 - 4.53 ) | 2.16 ( 4.35 ) | 2.16 ( 1.16 ) | 1.11 ( 0.09 ) |
| Acute Respiratory Failure | 7 | 2.44 ( 1.16 - 5.12 ) | 2.44 ( 5.94 ) | 2.44 ( 1.31 ) | 1.29 ( 0.26 ) |
| Cholecystectomy | 7 | 6.16 ( 2.94 - 12.94 ) | 6.16 ( 30.2 ) | 6.15 ( 3.31 ) | 2.62 ( 1.6 ) |
| Lower Respiratory Tract Infection Fungal | 6 | 112.79 ( 49.99 - 254.51 ) | 112.71 ( 642.68 ) | 109.07 ( 55.21 ) | 6.77 ( 5.66 ) |
| Ear Pain | 6 | 2.23 ( 1 - 4.97 ) | 2.23 ( 4.08 ) | 2.23 ( 1.14 ) | 1.16 ( 0.07 ) |
| Device Related Infection | 6 | 3.32 ( 1.49 - 7.39 ) | 3.32 ( 9.69 ) | 3.31 ( 1.7 ) | 1.73 ( 0.64 ) |
| Bronchoscopy | 6 | 170.14 ( 74.9 - 386.51 ) | 170.03 ( 959.42 ) | 161.85 ( 81.46 ) | 7.34 ( 6.22 ) |
| Bacterial Disease Carrier | 6 | 53.97 ( 24.08 - 120.94 ) | 53.93 ( 306.77 ) | 53.09 ( 27.03 ) | 5.73 ( 4.63 ) |
| Atypical Mycobacterial Lower Respiratory Tract Infection | 6 | 418.27(178.96 - 977.59 ) | 417.98 ( 2218.6 ) | 371.65 ( 182.66 ) | 8.54 ( 7.38 ) |
| Bronchiectasis | 6 | 5.45 ( 2.45 - 12.14 ) | 5.45 ( 21.75 ) | 5.44 ( 2.78 ) | 2.44 ( 1.35 ) |
| Nasal Polyps | 6 | 8.92 ( 4 - 19.88 ) | 8.91 ( 42.04 ) | 8.89 ( 4.55 ) | 3.15 ( 2.06 ) |
| Mycobacterial Infection | 6 | 22.18 ( 9.94 - 49.52 ) | 22.17 ( 120.49 ) | 22.03 ( 11.25 ) | 4.46 ( 3.37 ) |
| Burkholderia Cepacia Complex Infection | 6 | 132.96 ( 58.78 - 300.73 ) | 132.87 ( 755.25 ) | 127.83 ( 64.57 ) | 7 ( 5.88 ) |
| Appendicitis | 6 | 4.66 ( 2.09 - 10.38 ) | 4.66 ( 17.22 ) | 4.65 ( 2.38 ) | 2.22 ( 1.13 ) |

Abbreviation: ROR, reporting odds ratio; PRR, proportional reporting ratio; EBGM, empirical Bayesian geometric mean; EBGM05, the lower limit of the 95% CI of EBGM; IC, information component; IC025, the lower limit of the 95% CI of the IC; CI, confidence interval; PT,preferred term.

**Supplementary Table 4:**

Top 30 most common positive adverse events of Symdeko in males at the PT level

| PT | Case numbers | ROR(95%CI) | PRR(χ^2^) | EBGM(EBGM05) | IC(IC025) |
| --- | --- | --- | --- | --- | --- |
| Infective Pulmonary Exacerbation Of Cystic Fibrosis | 463 | 1468.23(1308.16 - 1647.89 ) | 1272.12(405147.04 ) | 876.6 ( 795.89 ) | 9.78 ( 9.61 ) |
| Hospitalisation | 267 | 23.89 ( 21.08 - 27.08 ) | 22.13 ( 5362.95 ) | 21.96 ( 19.78 ) | 4.46 ( 4.27 ) |
| Infection | 115 | 15.54 ( 12.9 - 18.72 ) | 15.06 ( 1504.38 ) | 14.98 ( 12.82 ) | 3.91 ( 3.63 ) |
| Pneumonia | 99 | 4.92 ( 4.03 - 6.01 ) | 4.81 ( 299.72 ) | 4.8 ( 4.06 ) | 2.26 ( 1.97 ) |
| Cough | 78 | 5.13 ( 4.1 - 6.43 ) | 5.04 ( 253.32 ) | 5.03 ( 4.17 ) | 2.33 ( 2 ) |
| Headache | 72 | 3.12 ( 2.47 - 3.94 ) | 3.08 ( 101.62 ) | 3.08 ( 2.53 ) | 1.62 ( 1.28 ) |
| Pulmonary Function Test Decreased | 68 | 200.33 ( 156.31 - 256.74 ) | 196.42 ( 12359.95 ) | 183.67 ( 149.24 ) | 7.52 ( 7.16 ) |
| Cystic Fibrosis Respiratory Infection Suppression | 66 | 827.41 ( 627.93 - 1090.28 ) | 811.67 ( 41483.81 ) | 630.3 ( 500.37 ) | 9.3 ( 8.91 ) |
| Cystic Fibrosis | 60 | 128.69 ( 99.14 - 167.04 ) | 126.48 ( 7149.16 ) | 121.09 ( 97.34 ) | 6.92 ( 6.54 ) |
| Nausea | 53 | 1.83 ( 1.4 - 2.4 ) | 1.82 ( 19.7 ) | 1.82 ( 1.45 ) | 0.86 ( 0.47 ) |
| Haemoptysis | 49 | 23.91 ( 18.01 - 31.74 ) | 23.59 ( 1051.58 ) | 23.4 ( 18.46 ) | 4.55 ( 4.14 ) |
| Malaise | 48 | 2.48 ( 1.87 - 3.3 ) | 2.46 ( 41.86 ) | 2.46 ( 1.94 ) | 1.3 ( 0.88 ) |
| Dyspnoea | 44 | 1.48 ( 1.1 - 1.99 ) | 1.47 ( 6.69 ) | 1.47 ( 1.15 ) | 0.56 ( 0.12 ) |
| Nasopharyngitis | 41 | 4.79 ( 3.52 - 6.51 ) | 4.74 ( 121.17 ) | 4.74 ( 3.66 ) | 2.24 ( 1.8 ) |
| Influenza | 40 | 6.88 ( 5.03 - 9.4 ) | 6.81 ( 198.13 ) | 6.8 ( 5.23 ) | 2.76 ( 2.31 ) |
| Abdominal Pain Upper | 38 | 4.37 ( 3.17 - 6.02 ) | 4.33 ( 97.5 ) | 4.33 ( 3.31 ) | 2.11 ( 1.65 ) |
| Lung Transplant | 36 | 128.69 ( 92 - 180.02 ) | 127.37 ( 4318.6 ) | 121.9 ( 92.05 ) | 6.93 ( 6.44 ) |
| Weight Decreased | 36 | 2.05 ( 1.48 - 2.85 ) | 2.04 ( 19.19 ) | 2.04 ( 1.55 ) | 1.03 ( 0.55 ) |
| Abdominal Pain | 27 | 2.48 ( 1.7 - 3.62 ) | 2.47 ( 23.64 ) | 2.47 ( 1.8 ) | 1.3 ( 0.75 ) |
| Decreased Appetite | 26 | 1.81 ( 1.23 - 2.66 ) | 1.8 ( 9.29 ) | 1.8 ( 1.3 ) | 0.85 ( 0.29 ) |
| Productive Cough | 22 | 6.71 ( 4.41 - 10.22 ) | 6.68 ( 106.06 ) | 6.66 ( 4.69 ) | 2.74 ( 2.13 ) |
| Depression | 22 | 2.21 ( 1.45 - 3.36 ) | 2.2 ( 14.49 ) | 2.2 ( 1.55 ) | 1.14 ( 0.54 ) |
| Hepatic Enzyme Increased | 22 | 7.18 ( 4.72 - 10.92 ) | 7.14 ( 115.89 ) | 7.12 ( 5.01 ) | 2.83 ( 2.23 ) |
| Sinusitis | 21 | 6.18 ( 4.02 - 9.49 ) | 6.15 ( 90.42 ) | 6.14 ( 4.28 ) | 2.62 ( 2 ) |
| Constipation | 20 | 1.73 ( 1.11 - 2.68 ) | 1.72 ( 6.09 ) | 1.72 ( 1.19 ) | 0.79 ( 0.15 ) |
| Sinus Operation | 19 | 158.71 ( 99.86 - 252.23 ) | 157.84 ( 2804.08 ) | 149.52 ( 101.47 ) | 7.22 ( 6.56 ) |
| Chest Discomfort | 19 | 4.09 ( 2.6 - 6.42 ) | 4.07 ( 44.06 ) | 4.07 ( 2.79 ) | 2.02 ( 1.38 ) |
| Treatment Noncompliance | 18 | 4.89 ( 3.07 - 7.77 ) | 4.87 ( 55.26 ) | 4.86 ( 3.3 ) | 2.28 ( 1.62 ) |
| Pseudomonas Infection | 17 | 27.29 ( 16.9 - 44.04 ) | 27.16 ( 424.27 ) | 26.91 ( 18.02 ) | 4.75 ( 4.06 ) |
| Viral Infection | 17 | 11.01 ( 6.83 - 17.74 ) | 10.96 ( 153.31 ) | 10.92 ( 7.32 ) | 3.45 ( 2.77 ) |

Abbreviation: ROR, reporting odds ratio; PRR, proportional reporting ratio; EBGM, empirical Bayesian geometric mean; EBGM05, the lower limit of the 95% CI of EBGM; IC, information component; IC025, the lower limit of the 95% CI of the IC; CI, confidence interval; PT,preferred term; AEs, adverse events.

**Supplementary Table 5**:

Top 30 most common positive adverse events of Symdeko in females at the PT level

| PT | Case numbers | ROR(95%CI) | PRR(χ^2^) | EBGM(EBGM05) | IC(IC025) |
| --- | --- | --- | --- | --- | --- |
| Infective Pulmonary Exacerbation Of Cystic Fibrosis | 713 | 1876.84  (1707.05-2063.52 ) | 1610.41  ( 763018.33 ) | 1071.67(989.93 ) | 10.07 ( 9.93 ) |
| Hospitalisation | 296 | 24.95 ( 22.17 - 28.07 ) | 23.53(6356.13 ) | 23.37 ( 21.18 ) | 4.55 ( 4.37 ) |
| Infection | 201 | 16.95 ( 14.72 - 19.53 ) | 16.32(2882.17 ) | 16.24 ( 14.43 ) | 4.02 ( 3.81 ) |
| Pneumonia | 141 | 5.96 ( 5.04 - 7.05 ) | 5.82 ( 565.2 ) | 5.82 ( 5.06 ) | 2.54 ( 2.29 ) |
| Headache | 129 | 2.24 ( 1.88 - 2.66 ) | 2.2 ( 85.78 ) | 2.2 ( 1.9 ) | 1.14 ( 0.88 ) |
| Cystic Fibrosis | 109 | 182.57(150.23 - 221.86 ) | 178.62(18237.33 ) | 169.2 (143.77 ) | 7.4 ( 7.12 ) |
| Cough | 94 | 3.71 ( 3.03 - 4.55 ) | 3.66 ( 182.57 ) | 3.66 ( 3.08 ) | 1.87 ( 1.57 ) |
| Dyspnoea | 83 | 1.84 ( 1.48 - 2.29 ) | 1.83 ( 31.49 ) | 1.83 ( 1.53 ) | 0.87 ( 0.55 ) |
| Haemoptysis | 82 | 53.17 ( 42.67 - 66.26 ) | 52.32(4062.76 ) | 51.5 (42.84 ) | 5.69 ( 5.36 ) |
| Cystic Fibrosis Respiratory Infection Suppression | 81 | 875.37(683.85-1120.54 ) | 861.26 (54843.53 ) | 678.85(552.14) | 9.41 ( 9.05 ) |
| Malaise | 78 | 2.04 ( 1.63 - 2.55 ) | 2.02 (40.45 ) | 2.02 ( 1.67 ) | 1.01 ( 0.69 ) |
| Pulmonary Function Test Decreased | 74 | 231.12(182.27 - 293.06 ) | 227.73(15595.64 ) | 212.67(174.35) | 7.73 ( 7.39 ) |
| Dizziness | 56 | 1.42 ( 1.09 - 1.85 ) | 1.42 ( 6.93 ) | 1.42 ( 1.14 ) | 0.5 ( 0.12 ) |
| Nasopharyngitis | 56 | 3.05 ( 2.35 - 3.97 ) | 3.03 ( 76.36 ) | 3.03 ( 2.43 ) | 1.6 ( 1.21 ) |
| Sinusitis | 50 | 4.59 ( 3.48 - 6.07 ) | 4.56 ( 138.99 ) | 4.55 ( 3.61 ) | 2.19 ( 1.78 ) |
| Influenza | 44 | 3.99 ( 2.96 - 5.37 ) | 3.96 ( 97.53 ) | 3.96 ( 3.09 ) | 1.98 ( 1.55 ) |
| Abdominal Pain Upper | 43 | 2.39 ( 1.77 - 3.23 ) | 2.38 ( 34.57 ) | 2.38 ( 1.85 ) | 1.25 ( 0.81 ) |
| Anxiety | 43 | 1.9 ( 1.41 - 2.57 ) | 1.89 ( 18.22 ) | 1.89 ( 1.47 ) | 0.92 ( 0.48 ) |
| Weight Decreased | 43 | 2.05 ( 1.52 - 2.77 ) | 2.04 ( 22.87 ) | 2.04 ( 1.59 ) | 1.03 ( 0.59 ) |
| Constipation | 43 | 2.42 ( 1.79 - 3.27 ) | 2.41 ( 35.45 ) | 2.41 ( 1.87 ) | 1.27 ( 0.83 ) |
| Chest Discomfort | 40 | 4.58 ( 3.35 - 6.25 ) | 4.55 ( 110.72 ) | 4.54 ( 3.5 ) | 2.18 ( 1.73 ) |
| Decreased Appetite | 38 | 2.05 ( 1.49 - 2.82 ) | 2.04 ( 20.2 ) | 2.04 ( 1.56 ) | 1.03 ( 0.56 ) |
| Depression | 34 | 2.13 ( 1.52 - 2.98 ) | 2.12 ( 20.11 ) | 2.12 ( 1.6 ) | 1.08 ( 0.59 ) |
| Productive Cough | 28 | 6.7 ( 4.62 - 9.72 ) | 6.67 ( 134.77 ) | 6.66 ( 4.88 ) | 2.73 ( 2.2 ) |
| Migraine | 26 | 2.39 ( 1.63 - 3.52 ) | 2.39 ( 20.98 ) | 2.39 ( 1.73 ) | 1.25 ( 0.7 ) |
| Lower Respiratory Tract Infection | 26 | 5.53 ( 3.76 - 8.13 ) | 5.51 ( 95.82 ) | 5.5 ( 3.98 ) | 2.46 ( 1.9 ) |
| Forced Expiratory Volume Decreased | 25 | 81.14 ( 54.5 - 120.79 ) | 80.74 ( 1920.46 ) | 78.78 ( 56.47 ) | 6.3 ( 5.73 ) |
| Pseudomonas Infection | 23 | 44.68 ( 29.58 - 67.49 ) | 44.48 ( 964.22 ) | 43.88 ( 31.07 ) | 5.46 ( 4.86 ) |
| Viral Infection | 23 | 8.35 ( 5.54 - 12.59 ) | 8.32 ( 147.79 ) | 8.3 ( 5.89 ) | 3.05 ( 2.46 ) |
| Treatment Noncompliance | 20 | 5.72 ( 3.69 - 8.88 ) | 5.7 ( 77.45 ) | 5.69 ( 3.94 ) | 2.51 ( 1.88 ) |

Abbreviation: ROR, reporting odds ratio; PRR, proportional reporting ratio; EBGM, empirical Bayesian geometric mean; EBGM05, the lower limit of the 95% CI of EBGM; IC, information component; IC025, the lower limit of the 95% CI of the IC; CI, confidence interval; PT,preferred term; AEs, adverse events.

**Supplementary Table 6**:

Top 30 most common positive adverse events of Symdeko in patients under 18 at the PT level

| PT | Case numbers | ROR(95%CI) | PRR(χ^2^) | EBGM(EBGM05) | IC(IC025) |
| --- | --- | --- | --- | --- | --- |
| Infective Pulmonary Exacerbation Of Cystic Fibrosis | 261 | 347.35(298.18 - 404.62 ) | 289.77( 54341.31 ) | 209.76 ( 184.61 ) | 7.71 ( 7.5 ) |
| Pneumonia | 68 | 11.99 ( 9.39 - 15.32 ) | 11.51 ( 645.56 ) | 11.36 ( 9.25 ) | 3.51 ( 3.15 ) |
| Hospitalisation | 66 | 23.49 ( 18.3 - 30.17 ) | 22.55 ( 1322.31 ) | 21.92 ( 17.79 ) | 4.45 ( 4.09 ) |
| Headache | 48 | 4.28 ( 3.21 - 5.71 ) | 4.18 ( 116.38 ) | 4.16 ( 3.27 ) | 2.06 ( 1.64 ) |
| Cough | 38 | 6.12 ( 4.43 - 8.46 ) | 6 ( 157.7 ) | 5.96 ( 4.55 ) | 2.58 ( 2.11 ) |
| Pulmonary Function Test Decreased | 36 | 107.53 ( 75.61 - 152.93 ) | 105.09 ( 3259.45 ) | 92.39 ( 68.81 ) | 6.53 ( 6.02 ) |
| Cystic Fibrosis Respiratory Infection Suppression | 33 | 151.68(104.09 - 221.03 ) | 148.51 ( 4041.99 ) | 124.29 ( 90.7 ) | 6.96 ( 6.42 ) |
| Cystic Fibrosis | 33 | 38.25 ( 26.87 - 54.44 ) | 37.46 ( 1116.51 ) | 35.74 ( 26.6 ) | 5.16 ( 4.65 ) |
| Nausea | 26 | 2.39 ( 1.62 - 3.52 ) | 2.37 ( 20.6 ) | 2.36 ( 1.71 ) | 1.24 ( 0.68 ) |
| Infection | 25 | 9.13 ( 6.14 - 13.59 ) | 9.01 ( 176.12 ) | 8.91 ( 6.39 ) | 3.16 ( 2.58 ) |
| Influenza | 24 | 7.55 ( 5.03 - 11.32 ) | 7.45 ( 132.89 ) | 7.38 ( 5.26 ) | 2.88 ( 2.3 ) |
| Weight Decreased | 23 | 6.21 ( 4.11 - 9.4 ) | 6.14 ( 98.36 ) | 6.1 ( 4.31 ) | 2.61 ( 2.01 ) |
| Constipation | 19 | 7.29 ( 4.63 - 11.49 ) | 7.22 ( 100.93 ) | 7.16 ( 4.89 ) | 2.84 ( 2.19 ) |
| Malaise | 16 | 3.02 ( 1.84 - 4.95 ) | 3 ( 21.3 ) | 2.99 ( 1.98 ) | 1.58 ( 0.88 ) |
| Abdominal Pain Upper | 16 | 3.82 ( 2.33 - 6.26 ) | 3.79 ( 32.8 ) | 3.78 ( 2.5 ) | 1.92 ( 1.21 ) |
| Decreased Appetite | 15 | 3.3 ( 1.98 - 5.49 ) | 3.28 ( 23.71 ) | 3.27 ( 2.13 ) | 1.71 ( 0.98 ) |
| Haemoptysis | 15 | 34.58 ( 20.56 - 58.16 ) | 34.26 ( 463.51 ) | 32.82 ( 21.25 ) | 5.04 ( 4.3 ) |
| Sinusitis | 15 | 12.53 ( 7.51 - 20.92 ) | 12.42 ( 155.11 ) | 12.24 ( 7.97 ) | 3.61 ( 2.88 ) |
| Nasopharyngitis | 14 | 3.82 ( 2.25 - 6.47 ) | 3.79 ( 28.69 ) | 3.78 ( 2.43 ) | 1.92 ( 1.17 ) |
| Pseudomonas Infection | 14 | 25.66 ( 15.03 - 43.82 ) | 25.44 ( 318.18 ) | 24.65 ( 15.76 ) | 4.62 ( 3.86 ) |
| Treatment Noncompliance | 14 | 6.84 ( 4.03 - 11.61 ) | 6.79 ( 68.59 ) | 6.74 ( 4.33 ) | 2.75 ( 2 ) |
| Abdominal Pain | 13 | 1.75 ( 1.01 - 3.02 ) | 1.74 ( 4.12 ) | 1.74 ( 1.1 ) | 0.8 ( 0.02 ) |
| Abdominal Discomfort | 12 | 5.99 ( 3.39 - 10.6 ) | 5.95 ( 49.13 ) | 5.91 ( 3.67 ) | 2.56 ( 1.76 ) |
| Dizziness | 11 | 2.09 ( 1.15 - 3.78 ) | 2.08 ( 6.18 ) | 2.08 ( 1.26 ) | 1.06 ( 0.22 ) |
| Alanine Aminotransferase Increased | 10 | 4.11 ( 2.2 - 7.66 ) | 4.09 ( 23.23 ) | 4.07 ( 2.42 ) | 2.03 ( 1.15 ) |
| Oropharyngeal Pain | 10 | 4.82 ( 2.58 - 8.99 ) | 4.79 ( 29.88 ) | 4.77 ( 2.83 ) | 2.25 ( 1.38 ) |
| Intestinal Obstruction | 10 | 20.5 ( 10.92 - 38.5 ) | 20.38 ( 179.5 ) | 19.87 ( 11.73 ) | 4.31 ( 3.43 ) |
| Sinus Operation | 9 | 110.38 ( 54.76 - 222.48 ) | 109.75 ( 846.96 ) | 95.97 ( 53.38 ) | 6.58 ( 5.61 ) |
| Viral Infection | 9 | 4.56 ( 2.37 - 8.8 ) | 4.54 ( 24.75 ) | 4.52 ( 2.61 ) | 2.18 ( 1.26 ) |
| Aspartate Aminotransferase Increased | 9 | 4.38 ( 2.27 - 8.45 ) | 4.36 ( 23.22 ) | 4.34 ( 2.51 ) | 2.12 ( 1.2 ) |

Abbreviation: ROR, reporting odds ratio; PRR, proportional reporting ratio; EBGM, empirical Bayesian geometric mean; EBGM05, the lower limit of the 95% CI of EBGM; IC, information component; IC025, the lower limit of the 95% CI of the IC; CI, confidence interval; PT,preferred term; AEs, adverse events.

**Supplementary Table 7**:

Top 30 most common positive adverse events of Symdeko in patients over 18 at the PT level

| PT | Case numbers | ROR(95%CI) | PRR(χ^2^) | EBGM(EBGM05) | IC(IC025) |
| --- | --- | --- | --- | --- | --- |
| Infective Pulmonary Exacerbation Of Cystic Fibrosis | 588 | 3393.57(3046.53-3780.15 ) | 2812.83(1049625.12 ) | 1786.54 ( 1632.34 ) | 10.8 ( 10.66 ) |
| Hospitalisation | 139 | 18.78 ( 15.84 - 22.26 ) | 18.06 ( 2236.97 ) | 18 ( 15.61 ) | 4.17 ( 3.92 ) |
| Pneumonia | 106 | 5.53 ( 4.56 - 6.71 ) | 5.39 ( 380.84 ) | 5.39 ( 4.58 ) | 2.43 ( 2.15 ) |
| Haemoptysis | 84 | 49.09 ( 39.49 - 61.02 ) | 47.92 ( 3823.29 ) | 47.46 ( 39.56 ) | 5.57 ( 5.25 ) |
| Headache | 82 | 2.41 ( 1.93 - 3 ) | 2.38 ( 65.92 ) | 2.37 ( 1.98 ) | 1.25 ( 0.93 ) |
| Cystic Fibrosis | 79 | 232.75 ( 185.27 - 292.38 ) | 227.42 ( 17019.18 ) | 217.36 ( 179.59 ) | 7.76 ( 7.43 ) |
| Infection | 69 | 8.74 ( 6.88 - 11.09 ) | 8.58 ( 462.45 ) | 8.57 ( 7.02 ) | 3.1 ( 2.75 ) |
| Dyspnoea | 66 | 2.02 ( 1.58 - 2.57 ) | 2 ( 33.13 ) | 2 ( 1.63 ) | 1 ( 0.64 ) |
| Cystic Fibrosis Respiratory Infection Suppression | 66 | 1383.66(1051.25-1821.17 ) | 1357.09 ( 70020.34 ) | 1062.68 ( 844.42 ) | 10.05 ( 9.66 ) |
| Cough | 62 | 3.69 ( 2.87 - 4.74 ) | 3.64 ( 119.25 ) | 3.64 ( 2.95 ) | 1.86 ( 1.5 ) |
| Nausea | 57 | 1.31 ( 1.01 - 1.7 ) | 1.3 ( 4.08 ) | 1.3 ( 1.05 ) | 0.38 ( 0 ) |
| Pulmonary Function Test Decreased | 47 | 190.66 ( 142.2 - 255.64 ) | 188.07 ( 8422.41 ) | 181.14 ( 141.73 ) | 7.5 ( 7.07 ) |
| Nasopharyngitis | 42 | 3.75 ( 2.76 - 5.08 ) | 3.71 ( 83.49 ) | 3.71 ( 2.88 ) | 1.89 ( 1.45 ) |
| Lung Transplant | 34 | 132 ( 93.74 - 185.87 ) | 130.71 ( 4262.73 ) | 127.33 ( 95.62 ) | 6.99 ( 6.5 ) |
| Abdominal Pain Upper | 33 | 2.93 ( 2.08 - 4.13 ) | 2.91 ( 41.53 ) | 2.91 ( 2.18 ) | 1.54 ( 1.04 ) |
| Influenza | 31 | 4.4 ( 3.09 - 6.27 ) | 4.37 ( 80.68 ) | 4.37 ( 3.25 ) | 2.13 ( 1.61 ) |
| Chest Discomfort | 27 | 4.27 ( 2.92 - 6.24 ) | 4.24 ( 67.04 ) | 4.24 ( 3.09 ) | 2.08 ( 1.54 ) |
| Constipation | 27 | 2.25 ( 1.54 - 3.28 ) | 2.24 ( 18.51 ) | 2.24 ( 1.63 ) | 1.16 ( 0.61 ) |
| Sinusitis | 25 | 4.06 ( 2.74 - 6.01 ) | 4.03 ( 57.13 ) | 4.03 ( 2.9 ) | 2.01 ( 1.44 ) |
| Forced Expiratory Volume Decreased | 23 | 77.29 ( 51.13 - 116.84 ) | 76.78 ( 1693.77 ) | 75.61 ( 53.51 ) | 6.24 ( 5.64 ) |
| Productive Cough | 21 | 6.09 ( 3.96 - 9.35 ) | 6.06 ( 88.65 ) | 6.05 ( 4.23 ) | 2.6 ( 1.98 ) |
| Abdominal Pain | 21 | 1.57 ( 1.02 - 2.42 ) | 1.57 ( 4.37 ) | 1.57 ( 1.1 ) | 0.65 ( 0.03 ) |
| Sinus Operation | 17 | 128.23 ( 79.13 - 207.78 ) | 127.6 ( 2081.12 ) | 124.38 ( 83.05 ) | 6.96 ( 6.27 ) |
| Nephrolithiasis | 14 | 5.48 ( 3.24 - 9.26 ) | 5.46 ( 50.98 ) | 5.45 ( 3.51 ) | 2.45 ( 1.7 ) |
| Dysphonia | 13 | 3.81 ( 2.21 - 6.56 ) | 3.79 ( 26.77 ) | 3.79 ( 2.4 ) | 1.92 ( 1.15 ) |
| Migraine | 13 | 2.43 ( 1.41 - 4.19 ) | 2.43 ( 10.91 ) | 2.43 ( 1.54 ) | 1.28 ( 0.51 ) |
| Gastrointestinal Disorder | 13 | 2.74 ( 1.59 - 4.72 ) | 2.73 ( 14.26 ) | 2.73 ( 1.73 ) | 1.45 ( 0.68 ) |
| Viral Infection | 13 | 8.07 ( 4.68 - 13.92 ) | 8.05 ( 80.12 ) | 8.03 ( 5.09 ) | 3.01 ( 2.23 ) |
| Alanine Aminotransferase Increased | 12 | 3.85 ( 2.18 - 6.78 ) | 3.84 ( 25.18 ) | 3.84 ( 2.39 ) | 1.94 ( 1.14 ) |
| Aspartate Aminotransferase Increased | 12 | 4.7 ( 2.67 - 8.29 ) | 4.69 ( 34.81 ) | 4.68 ( 2.91 ) | 2.23 ( 1.43 ) |

Abbreviation: ROR, reporting odds ratio; PRR, proportional reporting ratio; EBGM, empirical Bayesian geometric mean; EBGM05, the lower limit of the 95% CI of EBGM; IC, information component; IC025, the lower limit of the 95% CI of the IC; CI, confidence interval; PT,preferred term; AEs, adverse events.

**Supplementary Table 8**:

Top 50 most common positive adverse events of Symdeko excluding the most common concomitant drugs at the PT level

| PT | Case numbers | ROR(95%CI) | PRR(χ^2^) | EBGM(EBGM05) | IC(IC025) |
| --- | --- | --- | --- | --- | --- |
| Infective Pulmonary Exacerbation Of Cystic Fibrosis | 514 | 1152.29(1044.72-1270.94 ) | 1020.29( 453015.23 ) | 883.1 ( 813.58 ) | 9.79 ( 9.64 ) |
| Hospitalisation | 327 | 27.29 ( 24.38 - 30.55 ) | 25.37 ( 7647.83 ) | 25.28 ( 23 ) | 4.66 ( 4.49 ) |
| Headache | 122 | 2.9 ( 2.42 - 3.47 ) | 2.85 ( 147.7 ) | 2.85 ( 2.45 ) | 1.51 ( 1.25 ) |
| Infection | 107 | 9.96 ( 8.22 - 12.06 ) | 9.74 ( 840.14 ) | 9.73 ( 8.29 ) | 3.28 ( 3 ) |
| Pneumonia | 92 | 4.05 ( 3.29 - 4.97 ) | 3.98 ( 206.55 ) | 3.98 ( 3.35 ) | 1.99 ( 1.69 ) |
| Cough | 91 | 4.35 ( 3.54 - 5.36 ) | 4.28 ( 229.94 ) | 4.28 ( 3.6 ) | 2.1 ( 1.79 ) |
| Cystic Fibrosis | 81 | 155.22 ( 124.29 - 193.86 ) | 152.44 ( 11910.62 ) | 149 ( 123.71 ) | 7.22 ( 6.89 ) |
| Dyspnoea | 73 | 1.89 ( 1.5 - 2.38 ) | 1.87 ( 29.87 ) | 1.87 ( 1.54 ) | 0.9 ( 0.57 ) |
| Nausea | 67 | 1.28 ( 1.01 - 1.63 ) | 1.28 ( 4.08 ) | 1.28 ( 1.04 ) | 0.35 ( 0 ) |
| Pulmonary Function Test Decreased | 66 | 191.39 ( 149.58 - 244.88 ) | 188.58 ( 11971.71 ) | 183.34 ( 149.18 ) | 7.52 ( 7.16 ) |
| Haemoptysis | 62 | 34.1 ( 26.52 - 43.84 ) | 33.64 ( 1954.34 ) | 33.47 ( 27.13 ) | 5.06 ( 4.7 ) |
| Malaise | 62 | 2.08 ( 1.62 - 2.67 ) | 2.06 ( 34.14 ) | 2.06 ( 1.67 ) | 1.04 ( 0.68 ) |
| Cystic Fibrosis Respiratory Infection Suppression | 59 | 626.55 ( 478.99 - 819.56 ) | 618.31 ( 33231.45 ) | 565.14 ( 451.41 ) | 9.14 ( 8.75 ) |
| Nasopharyngitis | 45 | 3.26 ( 2.43 - 4.37 ) | 3.23 ( 69.63 ) | 3.23 ( 2.53 ) | 1.69 ( 1.27 ) |
| Abdominal Pain Upper | 41 | 2.96 ( 2.17 - 4.02 ) | 2.94 ( 52.53 ) | 2.94 ( 2.27 ) | 1.55 ( 1.11 ) |
| Influenza | 40 | 4.67 ( 3.42 - 6.37 ) | 4.63 ( 114.07 ) | 4.63 ( 3.57 ) | 2.21 ( 1.76 ) |
| Liver Function Test Increased | 39 | 18.12 ( 13.21 - 24.84 ) | 17.97 ( 623.59 ) | 17.92 ( 13.76 ) | 4.16 ( 3.7 ) |
| Weight Decreased | 38 | 1.91 ( 1.39 - 2.63 ) | 1.91 ( 16.41 ) | 1.9 ( 1.46 ) | 0.93 ( 0.47 ) |
| Sinusitis | 34 | 4.63 ( 3.3 - 6.48 ) | 4.6 ( 95.86 ) | 4.6 ( 3.47 ) | 2.2 ( 1.71 ) |
| Depression | 34 | 2.56 ( 1.83 - 3.59 ) | 2.55 ( 32.14 ) | 2.55 ( 1.92 ) | 1.35 ( 0.86 ) |
| Decreased Appetite | 33 | 1.95 ( 1.38 - 2.74 ) | 1.94 ( 15.11 ) | 1.94 ( 1.46 ) | 0.96 ( 0.46 ) |
| Constipation | 32 | 2.07 ( 1.46 - 2.93 ) | 2.06 ( 17.52 ) | 2.06 ( 1.54 ) | 1.04 ( 0.54 ) |
| Chest Discomfort | 31 | 4.46 ( 3.13 - 6.35 ) | 4.44 ( 82.65 ) | 4.44 ( 3.3 ) | 2.15 ( 1.64 ) |
| Productive Cough | 31 | 8.14 ( 5.72 - 11.59 ) | 8.09 ( 192.55 ) | 8.08 ( 6.01 ) | 3.01 ( 2.5 ) |
| Anxiety | 31 | 1.6 ( 1.12 - 2.28 ) | 1.6 ( 6.91 ) | 1.6 ( 1.19 ) | 0.67 ( 0.16 ) |
| General Physical Health Deterioration | 31 | 3.7 ( 2.6 - 5.27 ) | 3.68 ( 60.69 ) | 3.68 ( 2.74 ) | 1.88 ( 1.37 ) |
| Respiratory Symptom | 31 | 111.29 ( 77.94 - 158.91 ) | 110.53 ( 3309.14 ) | 108.71 ( 80.7 ) | 6.76 ( 6.25 ) |
| Lower Respiratory Tract Infection | 30 | 8.23 ( 5.75 - 11.79 ) | 8.19 ( 189.14 ) | 8.18 ( 6.05 ) | 3.03 ( 2.51 ) |
| Forced Expiratory Volume Decreased | 27 | 98.61 ( 67.36 - 144.37 ) | 98.03 ( 2554.94 ) | 96.6 ( 70.22 ) | 6.59 ( 6.04 ) |
| Abdominal Pain | 25 | 1.64 ( 1.11 - 2.43 ) | 1.64 ( 6.23 ) | 1.64 ( 1.18 ) | 0.71 ( 0.14 ) |
| Gastrointestinal Disorder | 25 | 3.71 ( 2.5 - 5.5 ) | 3.69 ( 49.15 ) | 3.69 ( 2.66 ) | 1.88 ( 1.32 ) |
| Lung Transplant | 23 | 76.98 ( 50.98 - 116.25 ) | 76.59 ( 1696.27 ) | 75.72 ( 53.64 ) | 6.24 ( 5.65 ) |
| Sinus Operation | 22 | 128.24 ( 84.01 - 195.75 ) | 127.61 ( 2711.01 ) | 125.2 ( 87.88 ) | 6.97 ( 6.36 ) |
| Pseudomonas Infection | 21 | 35.26 ( 22.94 - 54.2 ) | 35.1 ( 692.1 ) | 34.92 ( 24.37 ) | 5.13 ( 4.51 ) |
| Treatment Noncompliance | 21 | 5.58 ( 3.63 - 8.56 ) | 5.56 ( 78.46 ) | 5.55 ( 3.88 ) | 2.47 ( 1.86 ) |
| Hepatic Enzyme Increased | 21 | 4.21 ( 2.74 - 6.46 ) | 4.19 ( 51.1 ) | 4.19 ( 2.93 ) | 2.07 ( 1.45 ) |
| Nephrolithiasis | 20 | 5.93 ( 3.82 - 9.2 ) | 5.9 ( 81.46 ) | 5.9 ( 4.08 ) | 2.56 ( 1.93 ) |
| Viral Infection | 19 | 8.47 ( 5.39 - 13.29 ) | 8.44 ( 124.46 ) | 8.43 ( 5.78 ) | 3.08 ( 2.43 ) |
| Migraine | 18 | 2.55 ( 1.61 - 4.06 ) | 2.55 ( 16.95 ) | 2.55 ( 1.73 ) | 1.35 ( 0.69 ) |
| Alanine Aminotransferase Increased | 16 | 4.58 ( 2.8 - 7.49 ) | 4.57 ( 44.61 ) | 4.57 ( 3.03 ) | 2.19 ( 1.49 ) |
| Aspartate Aminotransferase Increased | 16 | 5.57 ( 3.41 - 9.11 ) | 5.56 ( 59.76 ) | 5.55 ( 3.68 ) | 2.47 ( 1.77 ) |
| Respiratory Tract Congestion | 15 | 15.07 ( 9.07 - 25.04 ) | 15.03 ( 195.99 ) | 14.99 ( 9.81 ) | 3.91 ( 3.18 ) |
| Therapy Cessation | 14 | 2.7 ( 1.6 - 4.57 ) | 2.7 ( 14.98 ) | 2.7 ( 1.74 ) | 1.43 ( 0.69 ) |
| Suicidal Ideation | 13 | 2.46 ( 1.42 - 4.23 ) | 2.45 ( 11.17 ) | 2.45 ( 1.55 ) | 1.29 ( 0.52 ) |
| Sinus Congestion | 12 | 14.29 ( 8.11 - 25.21 ) | 14.26 ( 147.66 ) | 14.23 ( 8.85 ) | 3.83 ( 3.03 ) |
| Respiratory Tract Infection | 12 | 6.12 ( 3.47 - 10.79 ) | 6.11 ( 51.25 ) | 6.1 ( 3.8 ) | 2.61 ( 1.81 ) |
| Lung Disorder | 12 | 3.39 ( 1.92 - 5.97 ) | 3.38 ( 20.11 ) | 3.38 ( 2.1 ) | 1.76 ( 0.95 ) |
| Surgery | 12 | 2.91 ( 1.65 - 5.14 ) | 2.91 ( 15.05 ) | 2.91 ( 1.81 ) | 1.54 ( 0.74 ) |
| Pneumothorax | 12 | 10.52 ( 5.97 - 18.55 ) | 10.5 ( 102.96 ) | 10.48 ( 6.52 ) | 3.39 ( 2.59 ) |
| Respiratory Failure | 12 | 2.63 ( 1.49 - 4.64 ) | 2.63 ( 12.12 ) | 2.63 ( 1.64 ) | 1.39 ( 0.59 ) |

Abbreviation: ROR, reporting odds ratio; PRR, proportional reporting ratio; EBGM, empirical Bayesian geometric mean; EBGM05, the lower limit of the 95% CI of EBGM; IC, information component; IC025, the lower limit of the 95% CI of the IC; CI, confidence interval; PT,preferred term; AEs, adverse events.
